# Predicting the risk of avian influenza A H7N9 infection in live-poultry markets across Asia

**DOI:** 10.1038/ncomms5116

**Published:** 2014-06-17

**Authors:** Marius Gilbert, Nick Golding, Hang Zhou, G. R. William Wint, Timothy P. Robinson, Andrew J. Tatem, Shengjie Lai, Sheng Zhou, Hui Jiang, Danhuai Guo, Zhi Huang, Jane P. Messina, Xiangming Xiao, Catherine Linard, Thomas P. Van Boeckel, Vincent Martin, Samir Bhatt, Peter W. Gething, Jeremy J. Farrar, Simon I. Hay, Hongjie Yu

**Affiliations:** 1 Biological Control and Spatial Ecology, Université Libre de Bruxelles, av FD Roosevelt 50, B-1050 Brussels, Belgium; 2 Fonds National de la Recherche Scientifique, rue d'Egmont 5, B-1000 Brussels, Belgium; 3 Spatial Ecology and Epidemiology Group, Department of Zoology, University of Oxford, Tinbergen Building, South Parks Road, Oxford OX1 3PS, UK; 4 Division of Infectious Disease, Key Laboratory of Surveillance and Early-warning on Infectious Disease, Chinese Center for Disease Control and Prevention, 155 Changbai Road, Changping District, 102206 Beijing, China; 5 Environmental Research Group Oxford, Department of Zoology, University of Oxford, South Parks Road, Oxford OX1 3PS, UK; 6 Livestock Systems and Environment (LSE), International Livestock Research Institute (ILRI), Old Naivasha Road, PO Box 30709, 00100 Nairobi, Kenya; 7 Fogarty International Center, National Institutes of Health, Bethesda, Maryland 20892, USA; 8 Department of Geography and Environment, University of Southampton, Highfield, Southampton SO17 1BJ, UK; 9 Scientific Data Center, Computer Network Information Center, Chinese Academy of Sciences, Zhongguancun Nansijie, Haidian District, 100190 Beijing, China; 10 Department of Microbiology and Plant Biology, Center for Spatial Analysis, University of Oklahoma, 101 David L. Boren Blvd, Norman, Oklahoma 73019, USA; 11 Institute of Biodiversity Sciences, Fudan University, 220A Handan Road, Yangpu District, Shanghai 200433, China; 12 Food and Agriculture Organization of the United Nations (FAO), Representation in Senegal, PO Box 3300, Dakar, Senegal; 13 Oxford University Clinical Research Unit–Wellcome Trust Major Overseas Unit, 190 Ben Ham Tu, District 5, Ho Chi Minh City, Vietnam

## Abstract

Two epidemic waves of an avian influenza A (H7N9) virus have so far affected China. Most human cases have been attributable to poultry exposure at live-poultry markets, where most positive isolates were sampled. The potential geographic extent of potential re-emerging epidemics is unknown, as are the factors associated with it. Using newly assembled data sets of the locations of 8,943 live-poultry markets in China and maps of environmental correlates, we develop a statistical model that accurately predicts the risk of H7N9 market infection across Asia. Local density of live-poultry markets is the most important predictor of H7N9 infection risk in markets, underscoring their key role in the spatial epidemiology of H7N9, alongside other poultry, land cover and anthropogenic predictor variables. Identification of areas in Asia with high suitability for H7N9 infection enhances our capacity to target biosurveillance and control, helping to restrict the spread of this important disease.

## Abstract (web summary)

An avian influenza virus of the H7N9 type, associated with live-poultry markets, has caused two human epidemics in China. Here, the authors develop a statistical model that predicts the risk of H7N9 infection in live-poultry markets across Asia, as a tool for disease surveillance and control.

## Graphical abstract

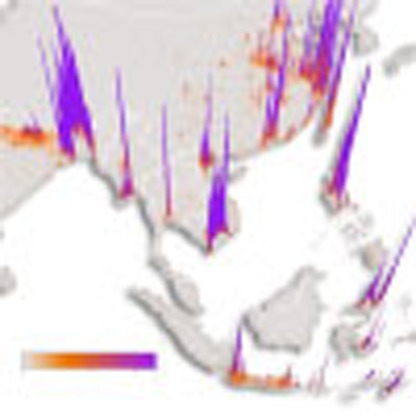

Despite the summer lull in reported human cases following its emergence in the spring of 2013, the resurgence of avian influenza A (H7N9) virus (hereafter ‘H7N9’) during the annual winter epidemic of seasonal influenza^1^,^2^ and recent revision of the importance of avian genetic lineages in past pandemics^3^ reaffirms concerns about its contemporary pandemic threat to global health^4^. To date, the majority of positive H7N9 isolations have been obtained from human, chicken or environmental samples that were directly or indirectly linked to live-poultry markets^5^,^6^,^7^,^8^. The diversity of genetic sequences from these samples suggests extensive and largely undetected spread of H7N9 in poultry preceding its appearance in markets and its ultimate infection in humans^9^. Investigations of other wild or domestic virus reservoirs remain inconclusive, however, confounding surveillance and control measures aimed at preventing its resurgence and further spread within and beyond China.

In addition to outbreak investigations and virus surveillance in humans and animals, predictive models linking the locations of the reported cases in humans and live-poultry markets to environmental risk factors can improve risk-based surveillance and control. This is demonstrated by the precedent of highly pathogenic avian influenza H5N1 virus, in which several epidemic waves in Asia were found to be strongly correlated with the spatial distributions of domestic ducks, human populations and wetlands^10^; these associations were then used to map the distribution of this disease^11^,^12^,^13^. Recent maps produced using data pooled from all historical cases closely matched these initial predictions^14^,^15^. These studies relied on geographically extensive case data, which were available only in the later stages of an epidemic. The ability to provide predictions of risk to new areas when few cases have been recorded would significantly improve immediate contingency planning. With the unfolding H7N9 epidemic, which many fear is limited to a fraction of its potential range, establishing the capacity of the model to extrapolate to other geographic regions is necessary to assess the utility of its predictions for decision making.

Predictive modelling of H7N9 in China and Asia is hampered by two problems. First, the distribution of H7N9 is strongly spatially clustered in the region of China in which it first appeared. The apparent accuracy of models derived from such initial data can be very high, as demonstrated by Fang *et al*.^16^ The ability of these models to make accurate predictions beyond this geographic range cannot, however, be quantified reliably through standard cross-validation procedures^17^. This is due to spatial correlation between test and training sets drawn from the same area and because the epidemic was limited to a small part of the potential geographic range. To address this issue, a geographic cross-validation would need to be implemented to quantitatively assess the models’ extrapolation capacity. Second, the lack of positive samples from active surveillance of poultry farms and the association of human and poultry cases with exposure to live-poultry markets^5^,^6^,^7^ suggest that markets, rather than birds, humans or poultry farms, are the appropriate unit for predictive analyses and surveillance.

Here we conduct an extensive country-wide census of live-poultry markets to allow market-level analysis, we update and improve high spatial resolution surfaces for poultry and human population distribution across China and we build a live-poultry market distribution model to allow extrapolation of our predictions to other areas across Asia. The models developed here with these new data can accurately predict the risk of infection of markets with H7N9 and have a good capacity to extrapolate their prediction geographically. Local live-poultry market density is the most important predictor variable of H7N9 infection risk at the market level. Other predictor variables of H7N9 infection risk include the population densities of chickens reared in extensive and intensive systems, water bodies, accessibility to major cities, human and domestic duck population density and rice land cover. The areas predicted to be most suitable for new H7N9 market infection include specific urban areas of China where the disease has not yet occurred, an extensive area in Bengal, the river deltas of Vietnam, and parts of Indonesia and Philippines.

## Results

### Distribution of H7N9 cases

**Figure 1:**
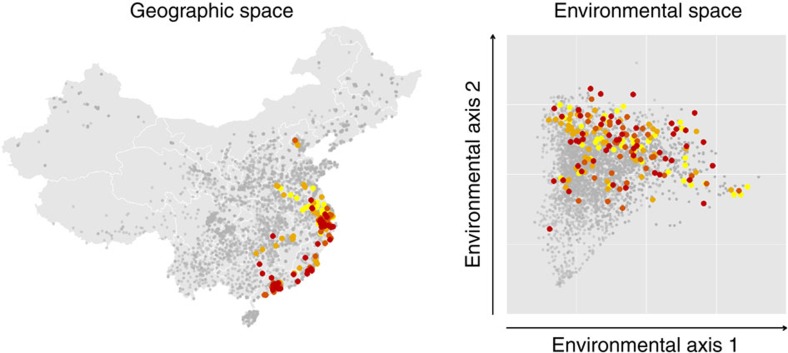
Distribution of potential H7N9-positive markets in mainland China in geographic and environmental space. In each panel, the distribution of H7N9-negative markets is shown by grey points. Potential H7N9-positive markets are shown by coloured points, with colours denoting the chronological order of cases. Colours range from yellow (earliest cases 19 February 2013) through light and dark orange to red (most recent cases 27 January 2014). Here environmental space is the Cartesian coordinate system defined by the first two principal components of environmental covariates at all market locations, which describe 56% of variation in the data set. The same pattern is apparent between other pairs of environmental axes, as illustrated in Supplementary Fig. 1.

Evaluation of the environmental space occupied by markets (Fig. 1), as determined by the values of key predictor variables for avian influenza (live-poultry market density, chicken, domestic duck and human population density, proportion of water and rice, and accessibility to major cities), showed that although infected markets were present in a limited area of geographic space, they covered a large portion of the available environmental space in China (Fig. 1). Furthermore, while the locations of newly infected markets spread steadily through geographical space, the environmental space occupied by infected markets was already wide from the early stages of the epidemic, and subsequent cases fell largely within the same environmental envelope (Fig. 1; Supplementary Fig. 1). This suggests that to date the environmental niche of the pathogen is fairly conserved and not expanding. As a corollary, predictive risk modelling based on the distribution of infected markets in environmental space could therefore be used to extrapolate to a much more extensive geographic area.

### Boosted regression tree models

An initial boosted regression tree (BRT) model predicting H7N9 presence/absence at the market level, fitted using the values of predictor variables at the same location as each market, provided a high predictive accuracy within the epidemic area (training data area under the curve (AUC)=0.964±0.001 s.e., standard cross-validation AUC=0.873±0.001 s.e.). Note that if instead of using live-poultry markets as units, we used the geographic pixel locations of infected markets and a set of randomly distributed pseudo-absences, a procedure similar to that used in previous modelling of H7N9 (ref. 16) and H5N1 (ref. 13), we obtained AUC values of 0.992 and 0.985 on the training and evaluation set, respectively. While those goodness of fit metrics indicate a high accuracy of prediction close to the training data, they provide little insight regarding the predictive capacity of the model to other geographical areas.

To provide a more accurate assessment of the model’s capacity to extrapolate to new regions, we carried out a disc-based geographical cross-validation procedure, dividing live-poultry markets into sets used to train the model (those within 1,000 km of a single, randomly-selected market) and those used to evaluate it (those more than 1,000 km from the market). Evaluated in this way, the market-level model had a fairly low geographic extrapolation capacity (mean AUC=0.515±0.026 s.e.). A second model was developed to account for the multiple values of environmental predictors in the area surrounding the market (emulating the aggregating effect of markets importing birds from a catchment area), using a Gaussian-weighted function. To determine the optimal value of the smoothing parameter *σ*, models were fitted with *σ*=0, 0.1, 0.3, 0.5, 0.7, 1.0 and 1.2 decimal degrees and their extrapolation capacity assessed using the spatially stratified cross-validation approach described above. The results of this procedure are shown in Supplementary Fig. 2. The extrapolation capacity of the model increased with larger *σ*, but reached a maximum at *σ*=0.7. This model produced a slightly less accurate fit to the training data (training data AUC=0.961±0.001 s.e., standard cross-validation AUC=0.913±0.001 s.e.), but a much improved capacity for geographical extrapolation (mean disc-based cross-validation AUC=0.745±0.025 s.e., Supplementary Fig. 3). Moreover, since the disc-based cross-validation statistics were evaluated on subsets of around one-third of the total number of infected markets, the true extrapolation capacity of the full model could be expected to be higher.

**Figure 2:**
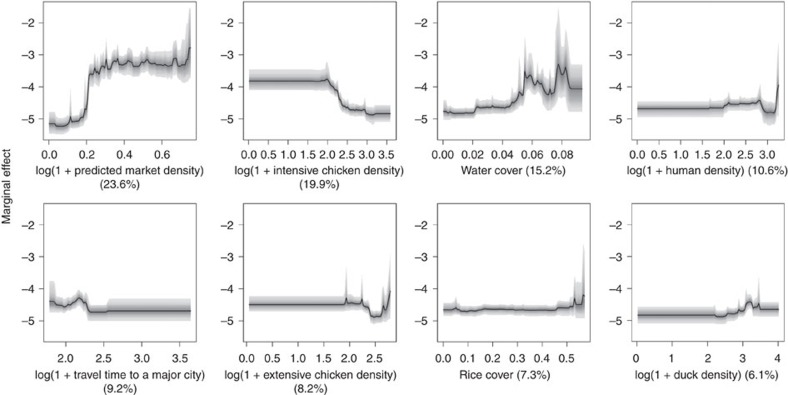
Marginal effect curves of each environmental predictor in the ensemble BRT model fitted to the full data set. The shaded areas represent the density of the predicted relationships to each environmental correlate (with the effect of the other correlates marginalized) from all 120 sub-models, within the lower and upper 95% quantiles of the distribution. The solid lines give the mean effect curves calculated from all models. Sub-plots are ordered by the mean of their RC to each sub-model, with these average RCs given in parentheses with each sub-plot.

Live-poultry market density was the most important predictor variable in the model (measured by its relative contribution (RC) to regression trees), with a strong positive association with H7N9 presence (Fig. 2). Other variables were, by decreasing order of their RC to the BRT ensemble, the population density of intensively raised chickens (RC 19.9%, negative association), the proportion of land covered by water (RC 15.1%, positive association), human population density (RC 10.6%, complex association), travel time to a major city (RC 9.2%, negative association), extensively raised chicken population density (RC 8.3%, complex association), the proportion of land covered by rice agriculture (RC 7.3%, positive association) and the population density of domestic ducks (RC 6.1%, positive association).

To predict risk outside China, it was necessary to generate a predictive map of live-poultry market density across Asia using a statistical model. To ensure that the infection risk predictions were not sensitive to the live-poultry market model, we constructed an additional model, equivalent to the model detailed above, but using the observed live-poultry market density as a covariate in place of modelled live-poultry market density. This model had a training data AUC of 0.960±0.004 s.e. and standard cross-validation AUC of 0.912±0.025 s.e., comparable to validation metrics for the model trained using the modelled live-poultry market density. The observed live-poultry market density had a mean RC of 24.8% to the ensemble model, a slightly higher value than the modelled live-poultry density used in the Asia-wide model (23.6%). The marginal effect curves for each of the covariates in this model were similar to those from the model with the modelled live-poultry market density, with a strong a positive association between H7N9 infection risk and live-poultry market density (Supplementary Fig. 4).

### Distribution of H7N9 predicted risk in Asia

**Figure 3:**
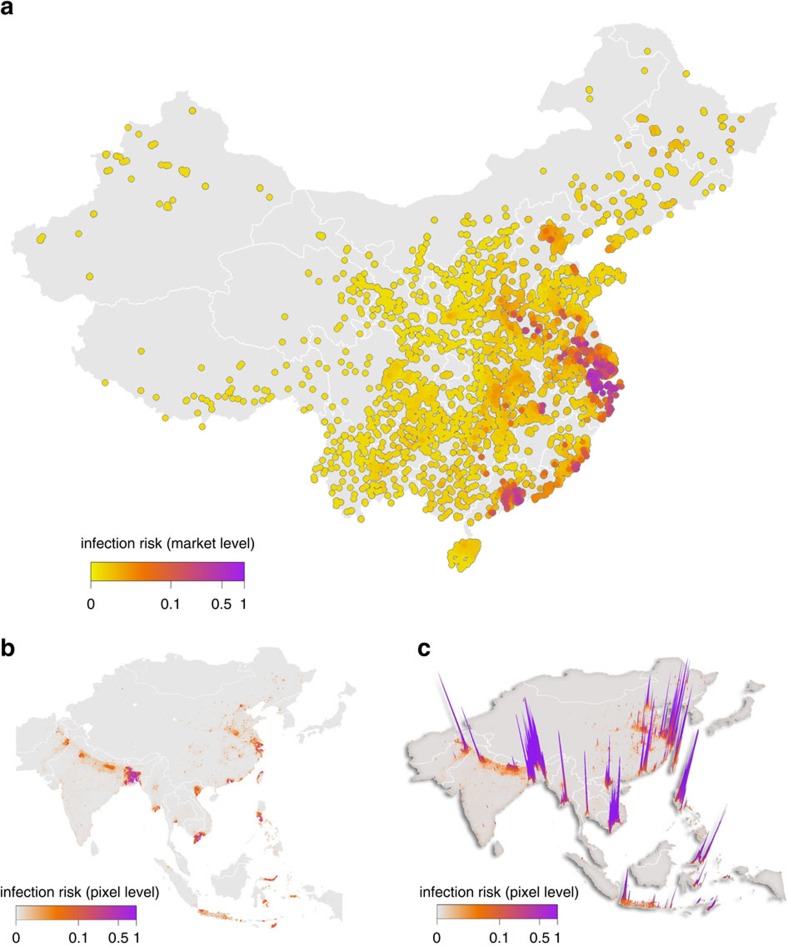
Geographic distribution of predicted H7N9 infection risk. (**a**) Market-level risk of H7N9 infection at live-poultry markets in mainland China; (**b**) pixel-level risk of H7N9 infection across Asia, the risk of at least one infected market being present in the given pixel; (**c**) a three-dimensional surface of the same data plotted in panel **b** with height representing infection risk to help illustrate its heterogeneity (see http://www.livestock.geo-wiki.org/ for a Google earth view). Note that infection risk is estimated as the probability that a market or pixel would be infected, if the average market-level infection prevalence in China were to remain constant. Since the pathogen is increasing in incidence, this number should instead be interpreted as a metric of infection risk; the relative probability of infection.

Using the predictive map of live-poultry market density for Asia, market-level H7N9 infection risk was converted into a metric of infection risk at the pixel level (analogous to the probability that at least one infected market is present in the pixel), and extrapolated to Southeast and South Asia (Fig. 3b,c). Pixel-level infection risk (on introduction) is predicted to be limited to peri-urban and urban areas, where live-poultry market density is the highest, and which themselves are characterized by the environmental risk factors highlighted above. For example, the greatest risk beyond already-infected areas is estimated to be in the Bengal regions of Bangladesh and India, the Mekong and Red river deltas in Vietnam and isolated parts of Indonesia and Philippines.

### Effect of data quality

To maximize the amount of data available to train the models, both markets which were indirectly associated with H7N9 infection (because these were the closest markets to the home location of a human case) as well as those which were directly associated with infection (either because a patient reported as visiting the market or because a positive sample was obtained from birds or the environment at that market) were considered to be infected with H7N9. To test our assumption that this was a reliable data set for training the model, we ran an additional full model using a data set in which markets were considered positive only if they were directly associated with H7N9 infection.

We compared this model with the full final model (using both indirectly and directly associated markets) by comparing the predicted infection risk maps and marginal effect curves of the two models. Note that it was not possible to compare validation statistics between these two models as they considered different data sets. Supplementary Figs 5 and 6 display the predicted risk of H7N9 infection at the market level and the pixel levels, respectively. In both cases, the spatial distribution of risk is very similar to that of the full model (Fig. 3). Note that the predicted risk is lower overall when using the data set containing only directly associated markets. This is an artefact of the absolute number of positive cases in the training set and is the reason that we consider relative, rather than absolute probability of infection here. Supplementary Fig. 7 shows the marginal effect curves of this model. The results are broadly comparable to those of the full final model (Fig. 2), with market density and water cover both appearing as major predictors, with similarly shaped curves, although the effect of intensively reared chickens is less than that in the full model.

## Discussion

Live-poultry market networks have previously been shown to be important in the spatial epidemiology of avian influenza in Asia, notably in the persistence and spread of highly pathogenic avian influenza H5N1 (refs 18, 19, 20, 21). The link between disease and markets appears to be even stronger for H7N9, as the main unit of infection observed has been the market^5^,^6^,^7^. Note, however, that if the unit of analysis in a model was a geographical area (administrative areas, for example, see ref. 16), an apparent effect of market density would be observed even if it had no epidemiological significance and equal individual probability of infection, since a region containing more markets is inherently more likely to contain at least one infected market. Because we considered infection risk at the market level, these analysis are unaffected by this bias. We found a strong positive association between market-level H7N9 presence and market density within an approximate radius of 70 km, which suggests a synergistic effect of high live-poultry market density in the neighbourhood of a given market.

Live-poultry markets bring together live birds from large catchment areas and unsold birds are commonly traded to other markets^20^,^21^,^22^; this results in market networks with numerous trade connections. The nature of the market network and of the characteristics of the markets composing the network (for example, time spent by animals in the market, cleaning operations, rest days’ schedules) influences the spread and persistence of disease over extended periods of time, even in the absence of re-introduction of viruses from poultry farms^19^. Higher densities of markets may exacerbate that risk and explain the strong spatial correlation with suitability for H7N9 infection. The epidemiological importance of dense networks of susceptible markets is also supported by the effectiveness of massive control efforts involving the closure of selected live-poultry markets, and the banning or regulation of trade in live poultry^8^. Characterizing the population size, species composition, trade volume and connectivity of live-poultry markets will be important to better quantify the role played by different types of markets in disease spread and persistence, hence allowing better targeting of control efforts.

The negative association between H7N9 presence and intensively raised chickens is likely explained by the absence of H7N9 cases in markets located in Northeastern China, an area where chicken production is highly intensive. Other covariates depict high suitability for H7N9 infection in the presence of intermediate to high proportions of land covered by water, peri-urban areas (intermediate travel time to city centres and intermediate human population density). These characteristics reflect the area where H7N9 first emerged, characterized by urban and peri-urban areas located within landscapes occupied by mixed extensive and intensive poultry farming in an environment rich in water and rice paddy fields.

Several sites key to the first investigations of H7N9 emergence share the above conditions (see a detailed map in Supplementary Fig. 8). The city of Huzhou in Zhejiang Province—where some of the highest H7N9 infection rates were also found in chicken and pigeon samples^7^—is surrounded by rice paddy fields that support free-ranging ducks and is <5 km from Taihu Lake, the largest freshwater lake in the Yangtze Delta plain near Shanghai. The nearby city of Hangzhou, located along the Qiantang river, where H7N9 was found in many markets^23^ together with a wide diversity of avian influenza subtypes^24^ is largely surrounded by rice paddy fields and other wetland-related agriculture. These sites were all predicted to be at relatively high risk of H7N9 infection (Fig. 3a). Interestingly, the more distant site of Poyang Lake was also predicted to be at relatively high risk (Fig. 3a). This site was identified as high risk by three previous studies of influenza A viruses: H5N1 in poultry^13^, H5N1 at the domestic/wild bird interface^25^ and H5N1 and H3N2 in humans^26^. A large diversity of avian influenza subtypes have been sampled in this area in both wild and sentinel ducks and at fairly high prevalences^27^; the area was recently the location of three reported human cases of novel avian influenza A (H10N8) virus infection in Nanchang, Jiangxi Province^28^. With genetic investigations indicating that H7N9 emerged from multiple reassortments of influenza viruses from domestic ducks, wild migratory duck and chickens^9^, these conditions common to all three sites indicate that the socioecological systems characterized by urban and peri-urban areas with their dense networks of live-poultry markets and surrounded by poultry farming in wetland-related agricultural landscapes may be high-risk areas or ‘hotspots’ for the emergence of new influenza viruses in humans.

The slow geographical expansion of the reported H7N9 cases in the central and southern provinces of China indicates that despite remarkably strict control efforts, H7N9 is difficult to contain along poultry market chains and may spread beyond the distribution indicated by the human cases, which have thus far been reported.

The pixel-level infection risk maps can be interpreted as a prediction of the potential geographic extent of live-poultry market infection in Asia, rather than which areas could be expected to be infected next. Generating robust spatiotemporal predictions of the geographic spread of infection would require data on connectivity between live-poultry markets, which is currently unavailable, as well as the development of approaches to integrate suitability models (such as that presented here) with models of pathogen dispersal. One should also note that these maps are influenced by the prediction of live-poultry market density, the output of a model that would benefit from live-poultry market census data unavailable today outside of China. Another important challenge for future research will be to assess the extent of H7N9 spread in poultry farming systems. The low pathogenicity of H7N9 in poultry makes it difficult to track through passive surveillance. Active surveillance will be needed to track the disease, assess the risk of human exposure and understand the RCs of farms, markets and wild birds to the disease reservoir during inter-epidemic periods. Given the high cost of active surveillance and the limited resources of some national veterinary services, risk-based prioritization of surveillance efforts is vital. No spatial model can provide a perfect geographical extrapolation, but the application of a robust spatial model with a prudent choice of sensitivity and specificity thresholds can facilitate evidence-based prioritization of surveillance in the region. This can help in early detection of new incursions, early response and active containment, minimizing impacts to agricultural livelihoods and reducing risk to human health.

## Methods

### H7N9 and live-poultry markets

All laboratory-confirmed human cases of influenza A (H7N9) virus infection in China are reported to the Chinese Center for Disease Control and Prevention (China CDC, Beijing, China) through a national surveillance system. Case definitions, surveillance for identification of cases of infection, and laboratory test assays have been described previously^6^. A joint team comprising staff from local or provincial CDC, or the China CDC, or both, carried out field investigations of the laboratory-confirmed human cases of influenza A (H7N9) virus infection. Demographic, epidemiological and basic clinical data on each laboratory-confirmed case of influenza A (H7N9) virus infection were obtained with standardized forms^5^,^8^,^29^, integrated into a database and used in this study, including geographic location and dates of illness onset for cases which were officially announced up until 2 February 2014. The National Health and Family Planning Commission determined that the collection of data from human influenza A (H7N9) cases was part of a continuing public health investigation of an emerging outbreak, and was therefore exempt from institutional review board assessment. In addition, a database of positive identification of influenza A (H7N9) in live-poultry markets was assembled, combining data from surveillance carried out by local CDC offices and the Ministry of Agriculture (MOA, http://www.moa.gov.cn/zwllm/yjgl/yqfb/), and with the records of influenza A (H7N9) published in scientific papers. This resulted in a data set containing the location and onset date of 284 human cases and 56 markets with documented evidence of influenza A (H7N9)-positive sampling (Supplementary Methods).

Four sources of data were combined to compile addresses of retail and wholesale live-poultry markets throughout China. The first was obtained from the official website of Ministry of Agriculture of China ( http://english.agri.gov.cn/) and Agricultural Bureaus at province and prefecture level. The second was the database of POI (point of interest) from the official gazetteer issued by National Administration of Surveying, Mapping and Geoinformation ( http://en.sbsm.gov.cn/). The third source resulted from data mining using search engines and social networks, by retrieving geo-features of searches with keywords related to live-poultry trading, such as ‘poultry’, ‘market’, ‘live poultry market’ and ‘farmers’ market’ both in Chinese and English. This source of data identified a number of recently established markets, which is yet to be incorporated into the formal data sets. Fourth, provincial Agricultural bureaus were also contacted to access unpublished data, from which the locations of around 1,000 live-poultry markets were obtained (<10% of all live-poultry markets). All together, this resulted in a database of 8,943 retail and wholesale poultry market locations.

The addresses of human H7N9 cases and live-poultry markets were geocoded through a complete and normalized address with five or six hierarchical administrative district names, then matched with gazetteer records. If this step failed, the Google geocoding service ( https://developers.google.com/maps/documentation/geocoding/) was used to locate the address.

Live-poultry markets were used as the unit of statistical analysis and were considered as potentially infected under the following conditions: (i) a confirmed influenza A (H7N9) human case reported a history of visit to that market (*n*=88); (ii) the market was nearest to a confirmed influenza A (H7N9) human case (*n*=123) or (iii) a positive influenza A (H7N9) sample was documented from that market (*n*=56). We excluded cases that were suspected to be human-to-human transmission because those infections may not have occurred through exposure to live-poultry markets. In total, 263 markets were assumed positive markets out of the entire data set of 8,943 retail and wholesale markets. The discrepancy between the total and the sum was due to some markets being identified as positive from multiple cases. A subset containing only the strongest evidences of market infection (conditions i and iii above) was also assembled.

### Risk factors

The analysis focused on a limited set of risk factors, including the density of live-poultry markets, in addition to a set of other factors that have been proven in the past to show consistent geographical correlation with avian influenza^14^. The set of risk factors included human population density (people km^−2^), a measure of accessibility (the travel time to a city of more than 50,000 habitants; minutes), duck density (birds km^−2^), chicken density (birds km^−2^), the proportion of land covered by open water (%), the proportion of land covered by rice cropping (%) and the predicted density of live-poultry markets (live-poultry markets km^−2^). Climatic factors were not considered in this analysis, because they have not been found to be consistently associated with avian influenza outbreaks in domestic poultry^14^ in geospatial terms. All risk factors were compiled at a spatial resolution of 0.083333 decimal degrees per pixel, corresponding to a spatial resolution of ~10 km × 10 km at the equator (Supplementary Methods). The human population density data layer was obtained from the WorldPop^30^ database in all countries where it was available to date, and from the Gridded Population of the World (GPW) database^31^ elsewhere (Supplementary Methods). The travel time to major cities was extracted from previously published accessibility maps^32^. Chicken and domestic duck density layers were produced using a revision of the method used in the Gridded Livestock of the World database^33^, described in Van Boeckel *et al*.^34^ and Prosser *et al*.^35^ and applied to an extensively improved data set with chicken and domestic duck census data compiled by China CDC (Supplementary Methods). Chicken density data were modified by disaggregating them into the densities of extensively and intensively raised chickens (Supplementary Methods). This was achieved by estimating the proportion of extensively raised chicken at the national level using national gross domestic product (GDP) *per capita*, distributing the total extensively raised chickens equally among the rural population with a fixed number of extensively raised chickens *per capita*, and estimating the density of intensively raised chickens by the difference between total chicken density and the density of extensively raised chickens at the pixel level. The proportion of area covered by water and rice was estimated from the Globcover land cover database^36^ and the Asia-wide rice maps^37^,^38^,^39^, respectively, by quantifying the proportion of pixels classified respectively as water and rice paddy field within each of the 0.083333 decimal degrees analysis pixel. The density of live-poultry markets was estimated in each pixel on the basis of a statistical model trained within China with the live-poultry market census data, and applied to other countries with corrections accounting for differences in absolute volume of poultry and for the proportion of poultry raised under extensive production systems (Supplementary Methods). The study area included China and all countries in Southern, Eastern and Southeastern Asia as defined by the United Nations Geoscheme. All predictor variables are displayed in the Supplementary Methods.

### Analysis

We used an ensemble BRT approach similar to that described in Bhatt *et al*.^40^ to establish a multivariate empirical relationship between the environmental suitability for H7N9 presence at the market level and the set of covariates sampled at each market location (Supplementary Methods). The BRT method has been shown to fit complicated response functions efficiently, while guarding against over-fitting, and has therefore been widely used for vector and disease distribution mapping^13^,^40^,^41^,^42^. Further, to reduce over-fitting and to account for sources of uncertainty in the data set, each model was an ensemble of 120 BRT sub-models, each fitted to a bootstrap sample of the data using the cross-validation algorithm of Elith *et al*.^41^ to select the optimal number of trees. We made the assumption that the risk posed to a market was dependent on risk factors in a catchment area surrounding the market. To account for this, each sub-model of the BRT ensemble was fitted to environmental covariates calculated using a weighted average of the covariate values surrounding each markets, with weights determined by a Gaussian kernel with s.d. *σ* representing the size of the catchment area. Key to our analyses was the assessment of the modelling extrapolation capacity over the geographic space. Standard cross-validation techniques generally divide the available data set in different folds, each containing a training set used to build the model and a validation set used to evaluate its predictive performances. However, because the selection of points for the training and validation sets is made at random, the resulting training and validation sets are rarely truly independent because of the frequent spatial clustering of cases^43^. This leads to overestimation of the goodness of fit compared with a comparison made with a truly independent validation set and can lead to selection of models with poor capacity to extrapolate to new areas. A disc-based spatial cross-validation procedure was implemented so that points forming the training set were geographically separated from the points of the evaluation set. For each cross-validation fold, a market was selected at random, and all markets within a radius of 1,000 km were designated as an evaluation set, whereas markets beyond that range were used as the model training set. Discs were placed at random, but subject to the constraint that at least 45 positive markets (around 28% of the total) were present in both the training and evaluation sets (see Supplementary Methods for details). This procedure was repeated five times using different sets of markets for model training and evaluation, and on each replicate goodness of fit was quantified by the AUC of the receiver operating characteristic plot. A range of values for the parameter *σ* (the range of the Gaussian kernel) was tested between 0.1 and 1.0 decimal degrees, and the value optimizing the goodness of fit of the disc-fold validation was used in the final model, using all of the available data. Two types of output maps were produced, showing the predicted market-level risk *P*_*i*_ for each pixel *i* in Asia (that is, the risk for an individual market if it were located in that pixel) and the combined risk at the pixel level 1−(1−*P*_*i*_)^*Ni*^, where *N*_*i*_ is the predicted number of live-poultry markets in pixel *i* (that is, the risk of at least one infected market being present in pixel *i*).

## Additional information

**How to cite this article:** Gilbert, M. *et al*. Predicting the risk of avian influenza A H7N9 infection in live-poultry markets across Asia. *Nat. Commun.* 5:4116 doi: 10.1038/ncomms5116 (2014).

## Disclaimer

The views expressed are those of the authors and do not necessarily represent the policy of the China CDC.

## Supplementary Material

Supplementary Information Supplementary Figures 1-15, Supplementary Table 1, Supplementary Methods and Supplementary References
