# Supplementary material for: Predicting the risk of avian influenza A H7N9 infection in live-poultry markets across Asia
Source: Nat Commun. 2014 Jun 17;5:4116. doi: 10.1038/ncomms5116 (PMC4061699; doi:10.1038/ncomms5116)
Supplement: Supplementary Information — Supplementary Figures 1-15, Supplementary Table 1, Supplementary Methods and Supplementary References [file ncomms5116-s1.pdf]

## SUPPLEMENTARY FIGURES

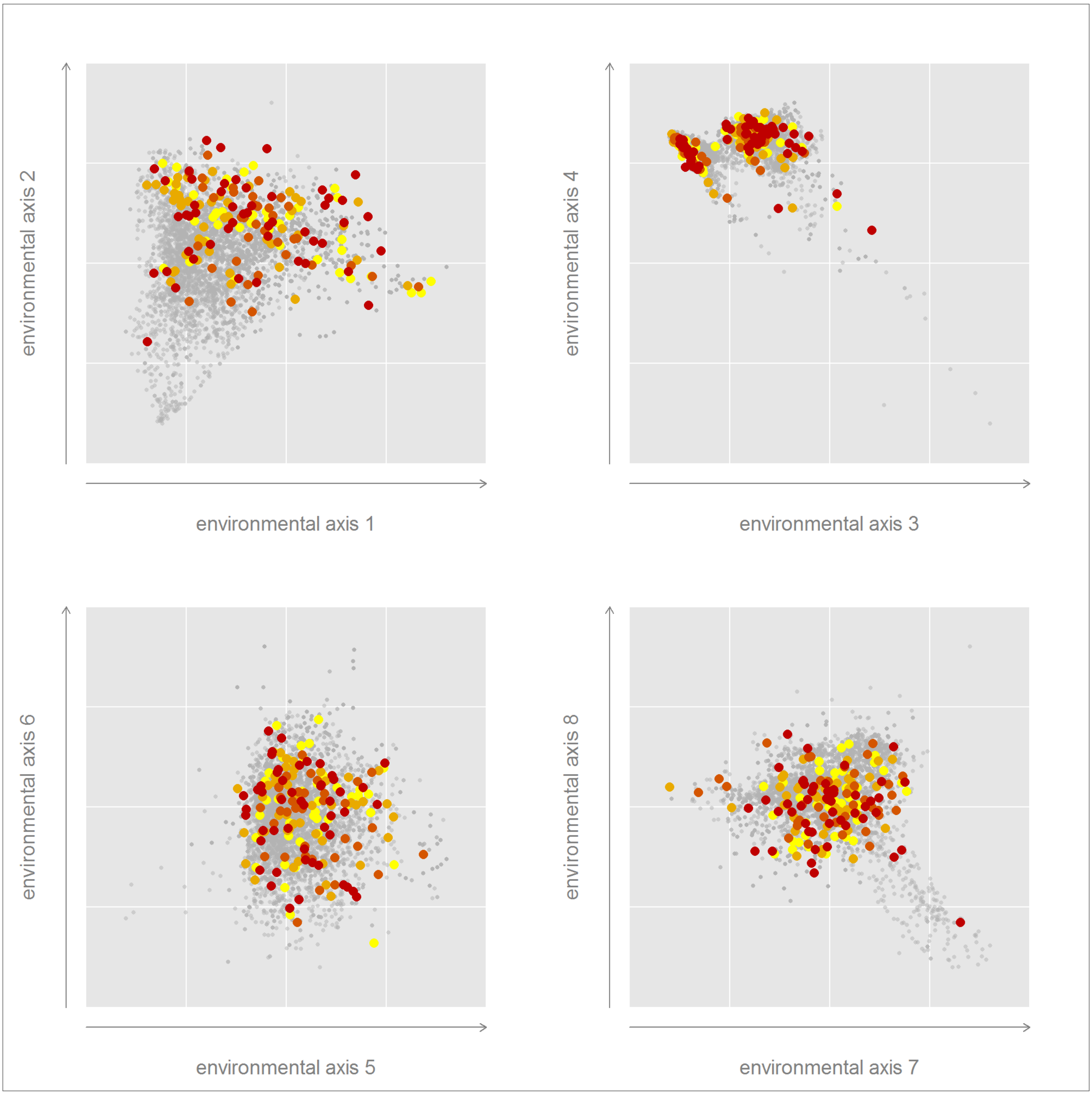


**Supplementary Figure 1. Distribution of potential H7N9 positive markets in China in environmental space.** Each panel is viewed from a different pair of environmental axes (principal components of the environmental covariates at all market locations). The distribution of H7N9 negative markets is shown by grey points. Potential H7N9 positive markets are shown by coloured points with colours denoting the chronological order of cases. Colours range from yellow (earliest cases) through light and dark orange to red (most recent cases).

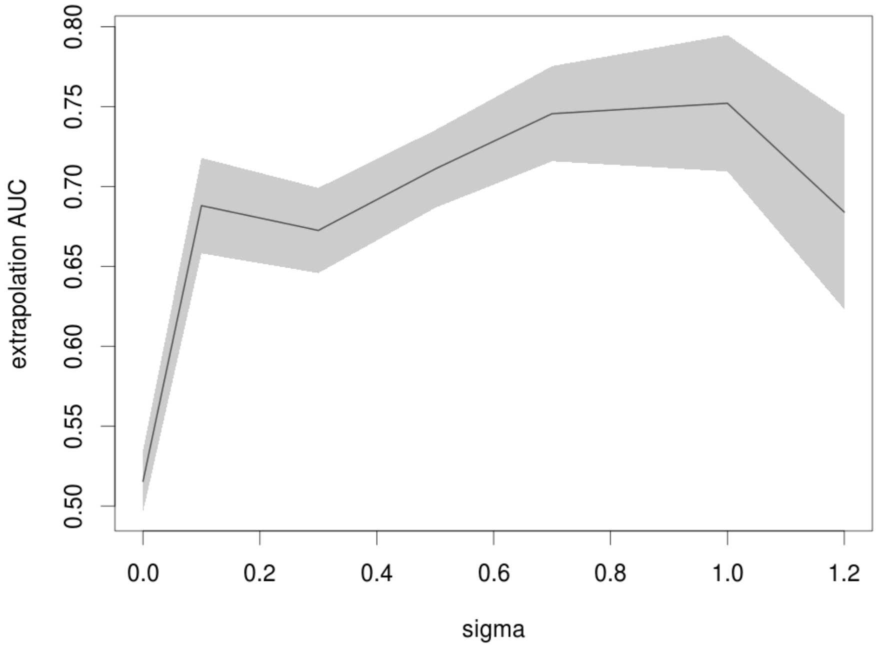


**Supplementary Figure 2. Disc-fold validation statistics for a range of values of the smoothing parameter σ.** The solid line gives the mean AUC and the shaded region gives ± 1 standard error.

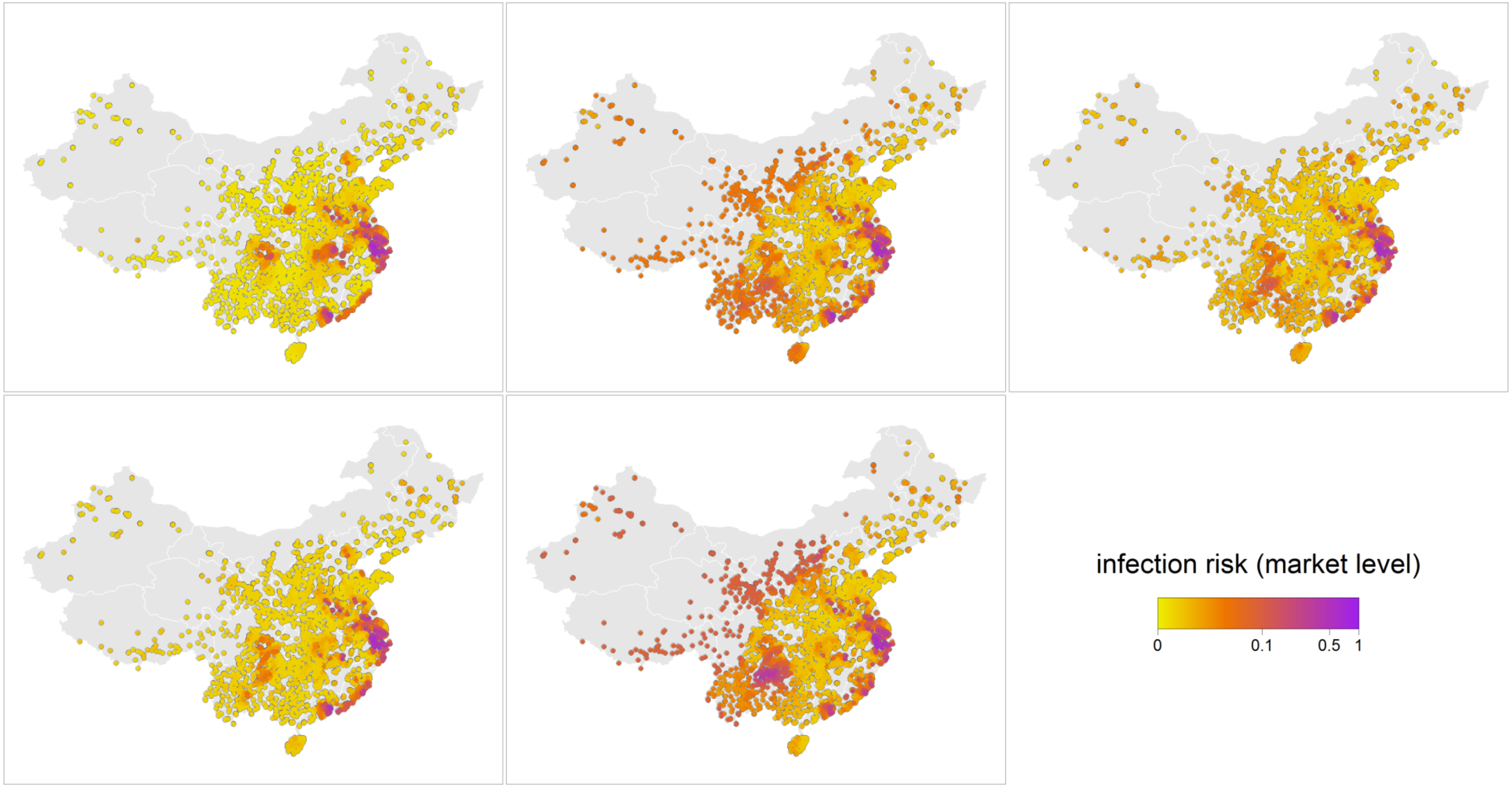


**Supplementary Figure 3. Predicted market-level infection risk in mainland China for models trained using each of the 5 disc-fold data subsets and the optimal smoothing parameter $\sigma = 0.7$.** The similarity of predictions between all five models, particularly in the east of the country illustrates that the environmental signature of infected markets is common across geographic space. A higher variability between the five models can be noted in the western part of the country.

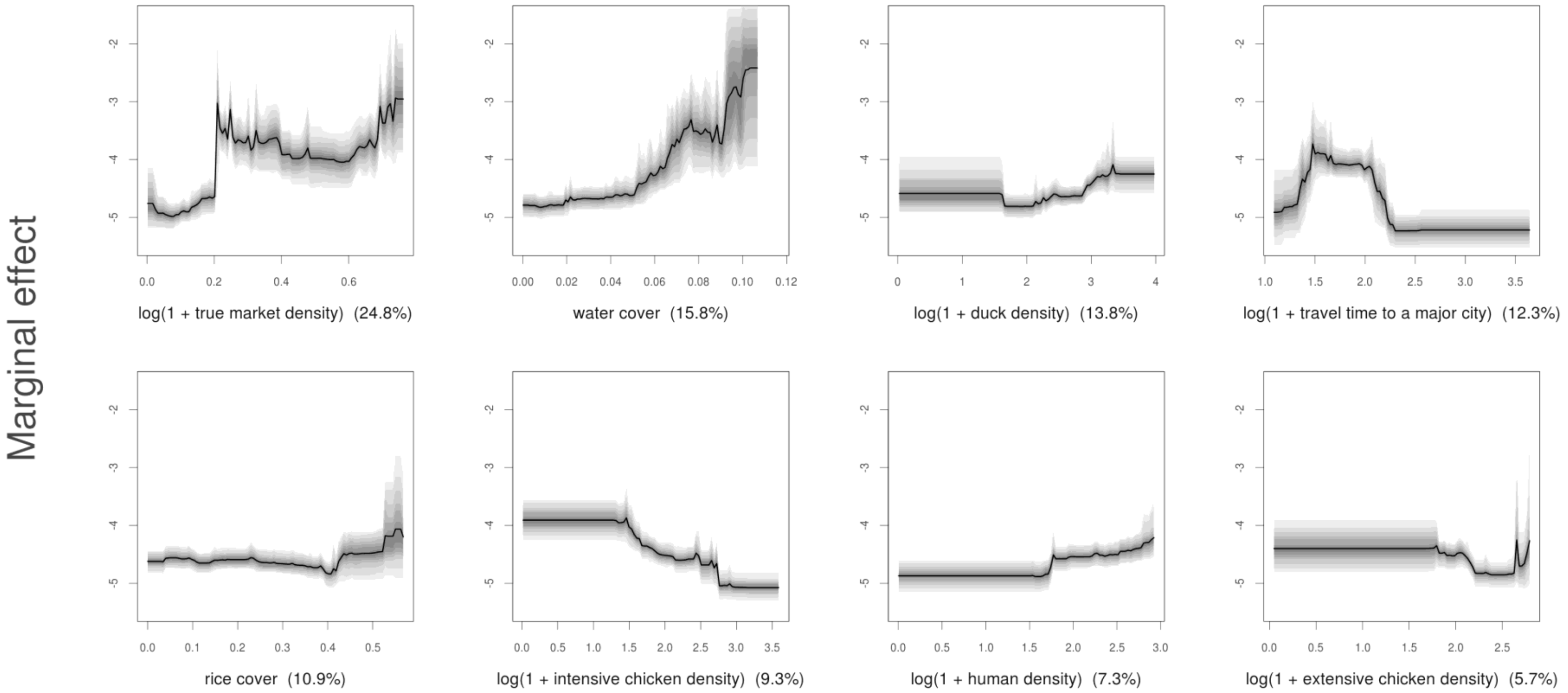


**Supplementary Figure 4. Marginal effect curves of each environmental predictor from a model trained with H7N9 positive markets incriminated using observed live-poultry market data.** The shaded areas represent the density of the predicted relationships to each environmental covariate (with the effect of the other correlates marginalized) from all 120 sub-models, within the lower and upper 95% quantiles of the distribution. The solid lines give the mean effect curves calculated from all models. Tick marks on the lower and upper inside edges of each sub-plot show the values of the predictor for H7N9 negative and potentially positive markets respectively. Sub-plots are ordered by the mean of their relative contribution to each sub-model, with these average relative contributions given in parentheses with each sub-plot.

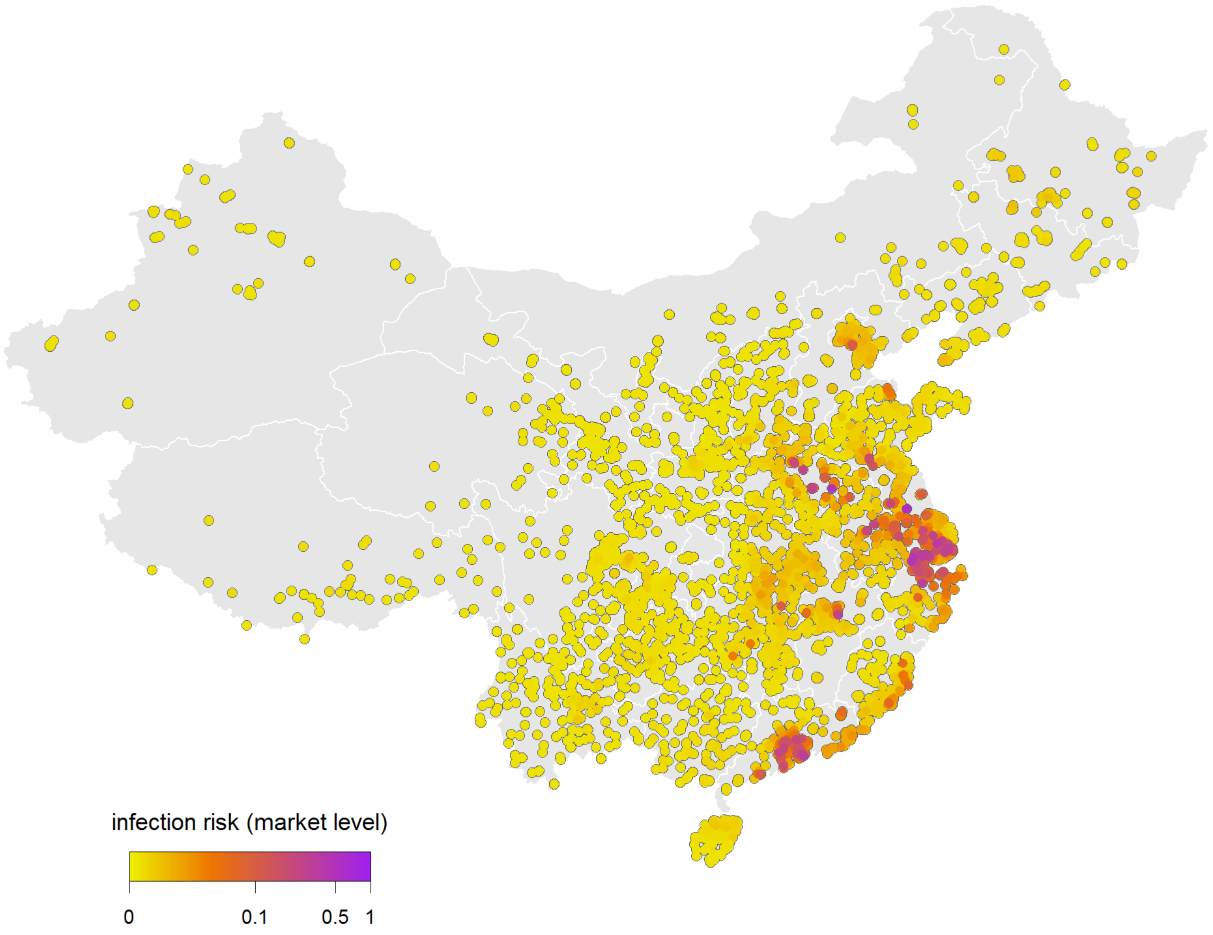


**Supplementary Figure 5. Predicted market-level infection risk at markets in mainland China for a model trained with H7N9 positive markets incriminated using only the high-quality data subset.**

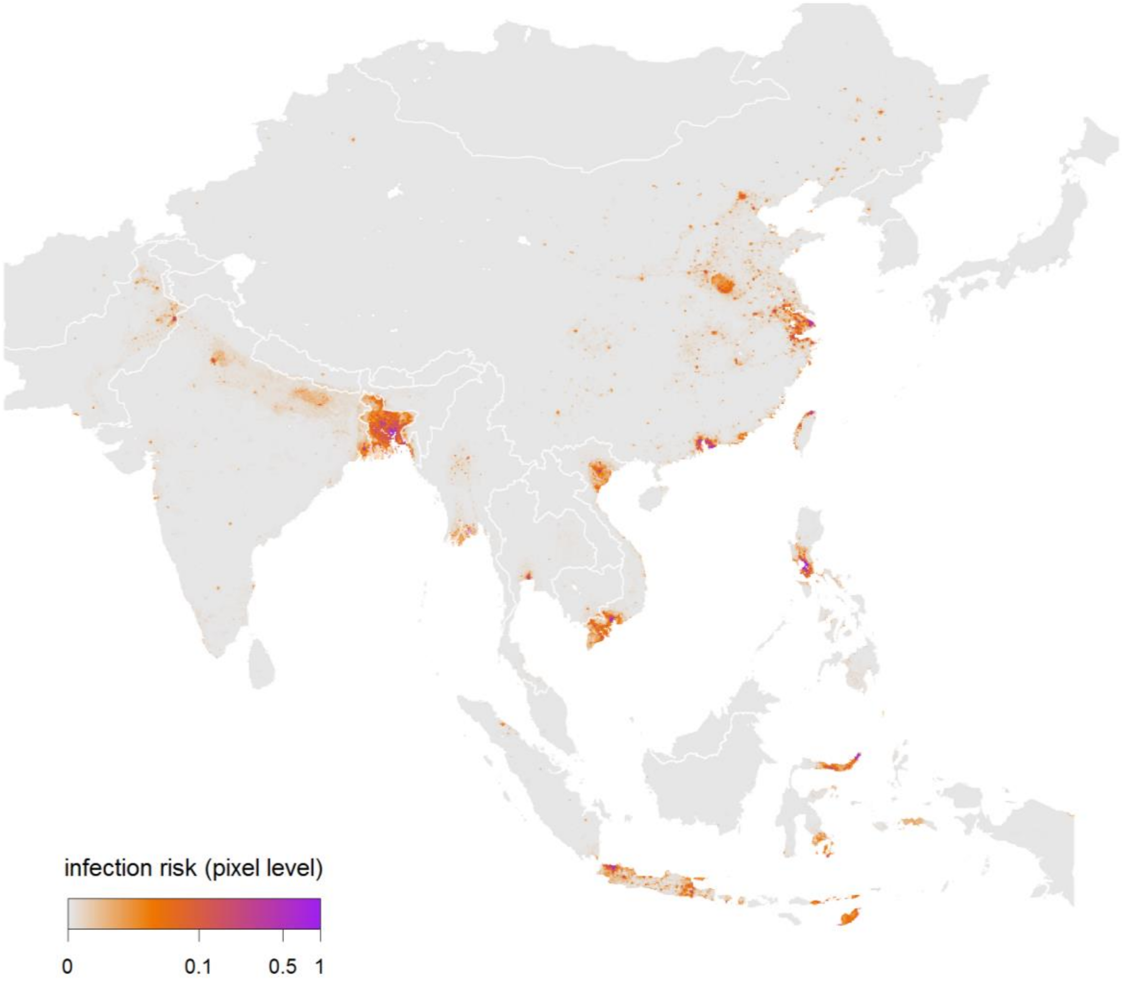


**Supplementary Figure 6. Predicted pixel-level infection risk across Asia from a model trained with H7N9 positive markets incriminated using only the high-quality data subset.**

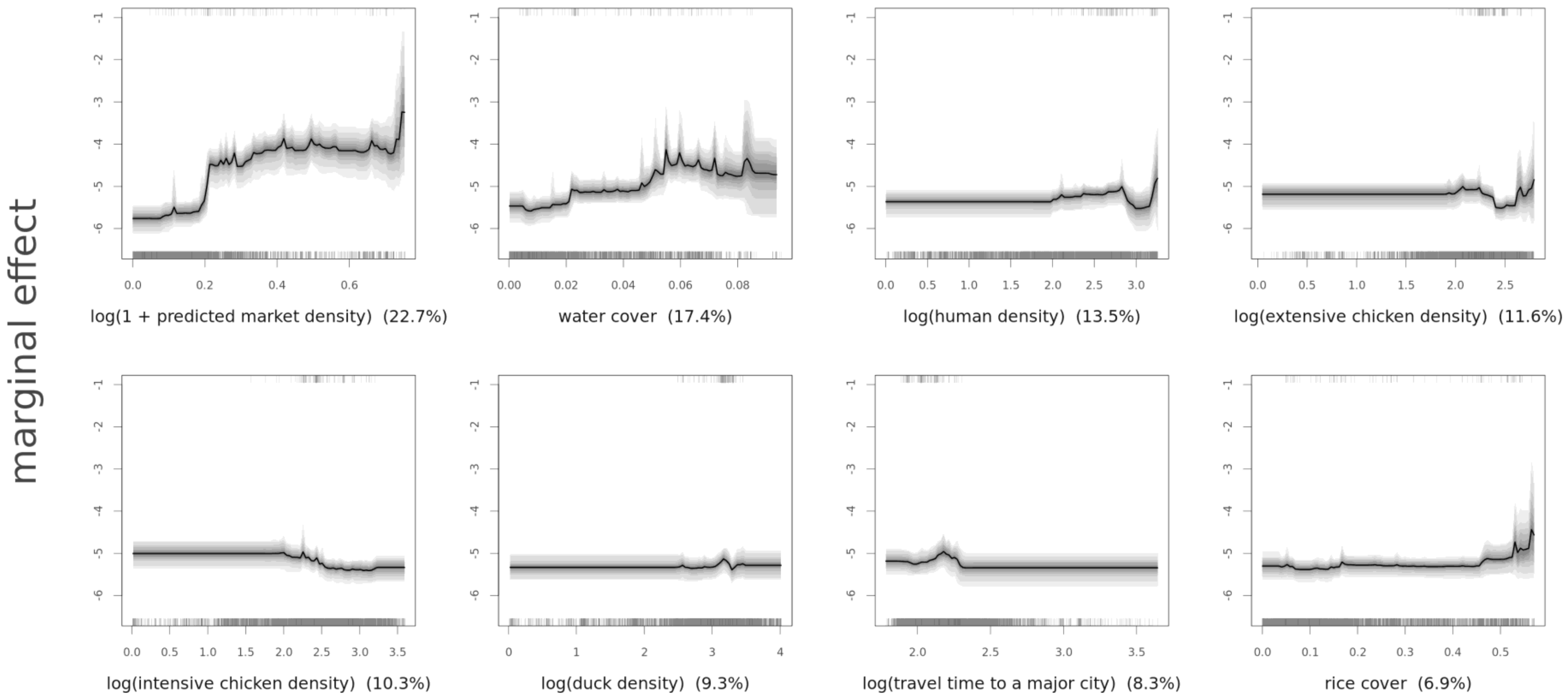


**Supplementary Figure 7. Marginal effect curves of each environmental predictor from a model trained with H7N9 positive markets incriminated using only the high-quality data subset.** The shaded areas represent the density of the predicted relationships to each environmental covariate (with the effect of the other correlates marginalized) from all 120 sub-models, within the lower and upper 95% quantiles of the distribution. The solid lines give the mean effect curves calculated from all models. Tick marks on the lower and upper inside edges of each sub-plot show the values of the predictor for H7N9 negative and potentially positive markets respectively. Sub-plots are ordered by the mean of their relative contribution to each sub-model, with these average relative contributions given in parentheses with each sub-plot.

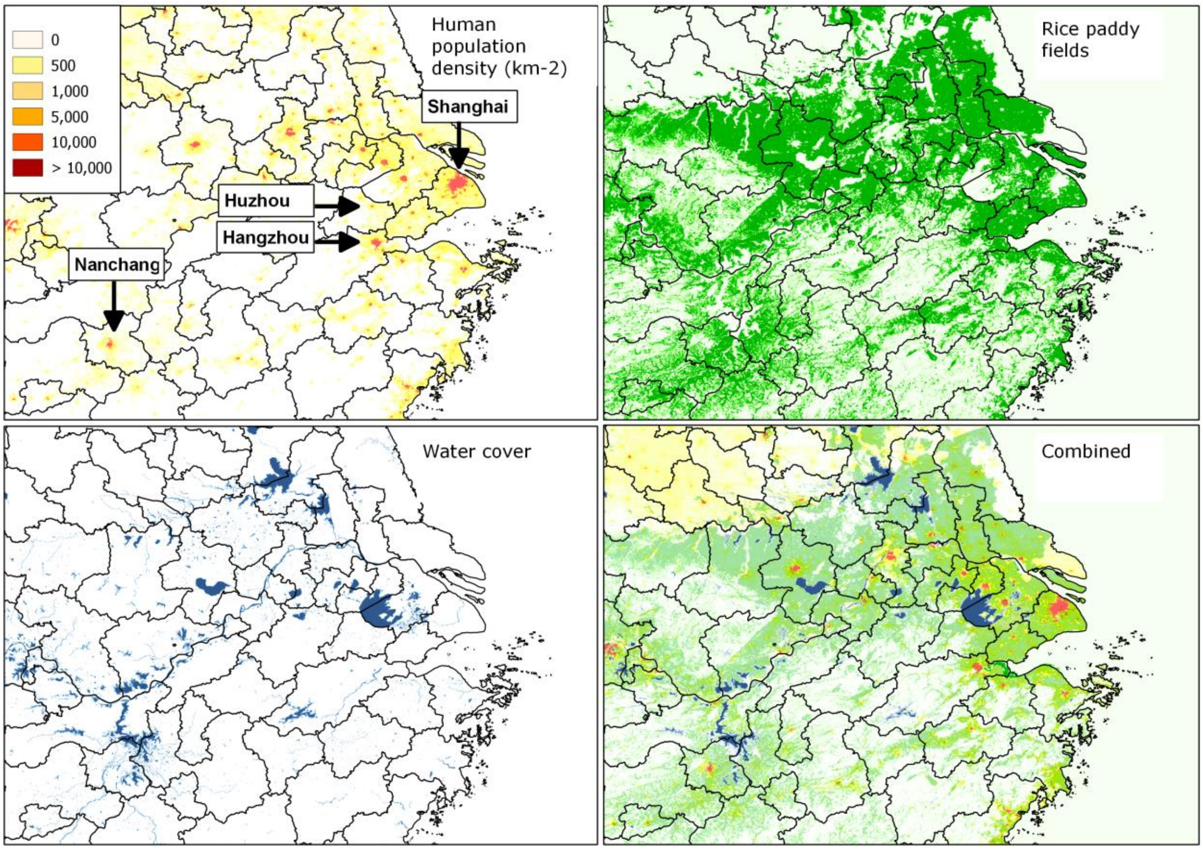


**Supplementary Figure 8. Distribution of key sites in relation to peri-urban and urban extents (top left), rice paddy fields land cover (top right), water land cover (bottom left), and combined with transparency (bottom right).**

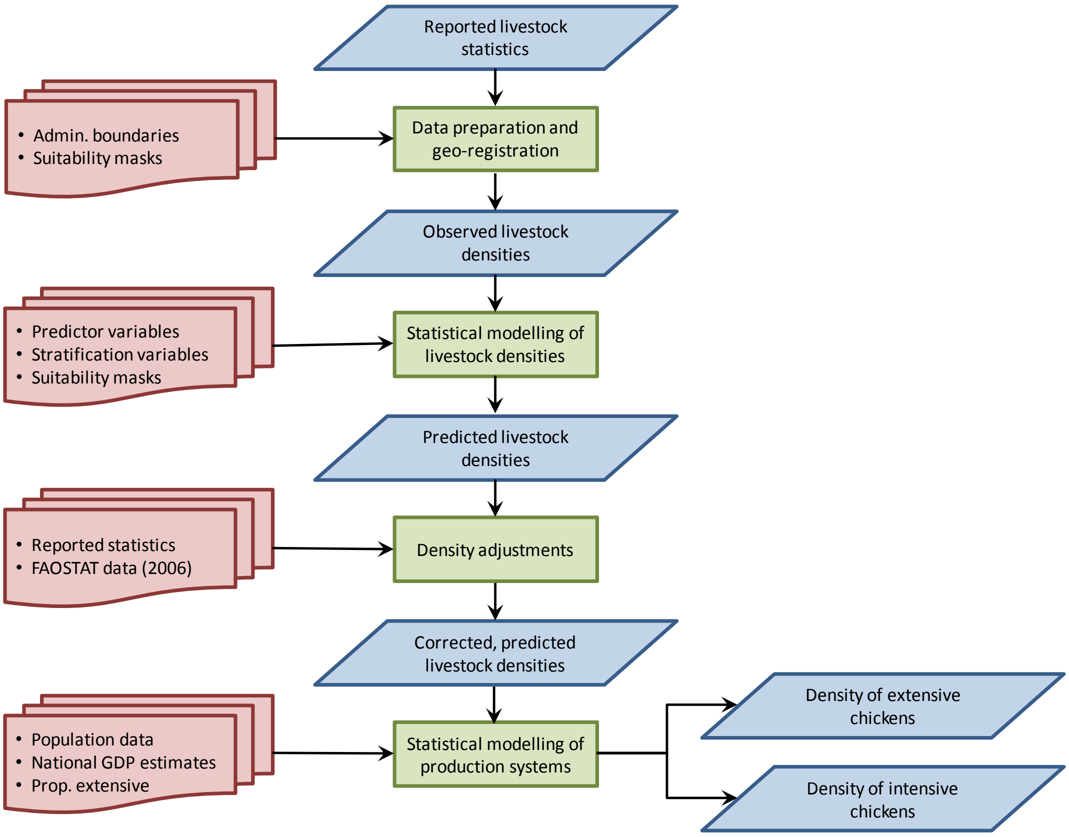


**Supplementary Figure 9. Schematic overview of the livestock modelling process used to downscale poultry census data.**

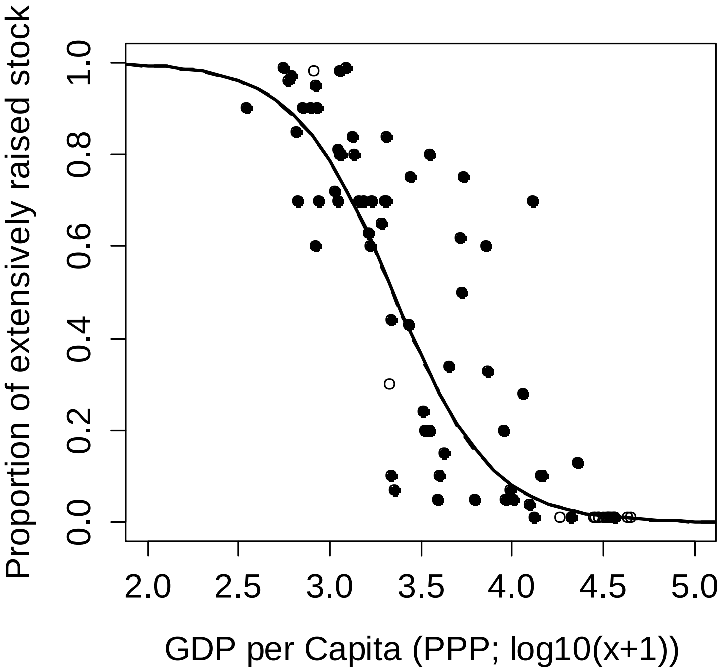


**Supplementary Figure 10. Relationship between the proportions of chickens raised in extensive conditions (PExt) and log per capita GDP from corresponding years (PPT 2006) for 79 countries (variable years) globally.** The solid line shows values of for 2006 estimated from the model presented in the supplementary methods.

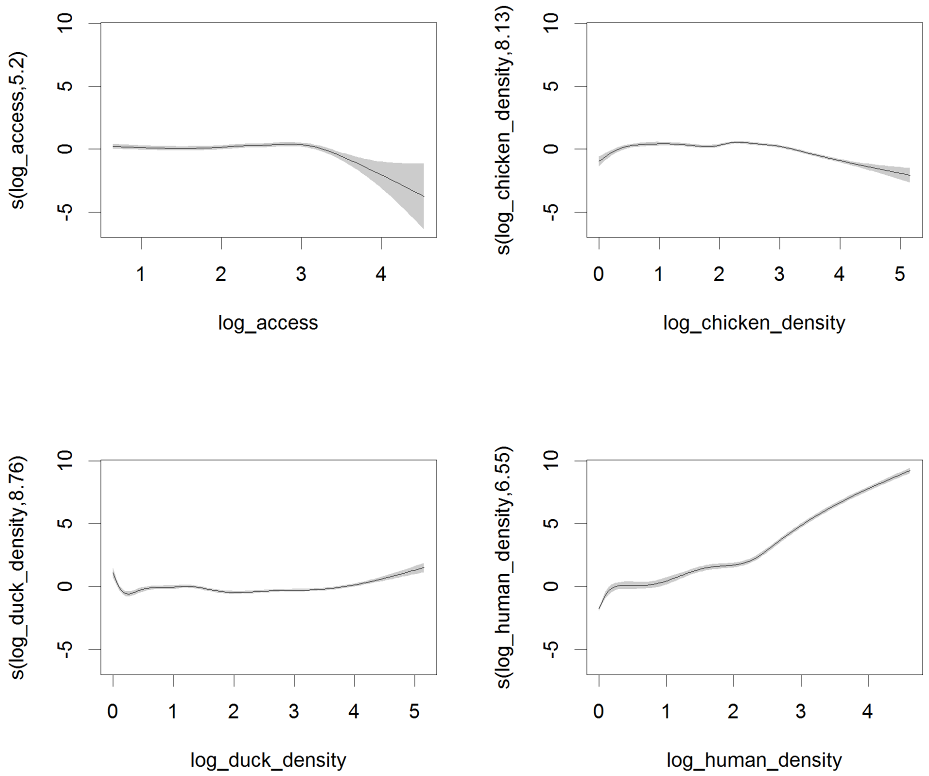


**Supplementary Figure 11. Smooth terms fitted in the final GAM live poultry market model.** Solid lines give the fitted term and the grey shaded areas are ± 2 standard errors. The Y axis gives the response of the smoother on the internal model scale. The dimension of the basis function used to fit the cubic regression spline is given in the Y axis labels, with larger numbers indicating a more complex fitted curve.

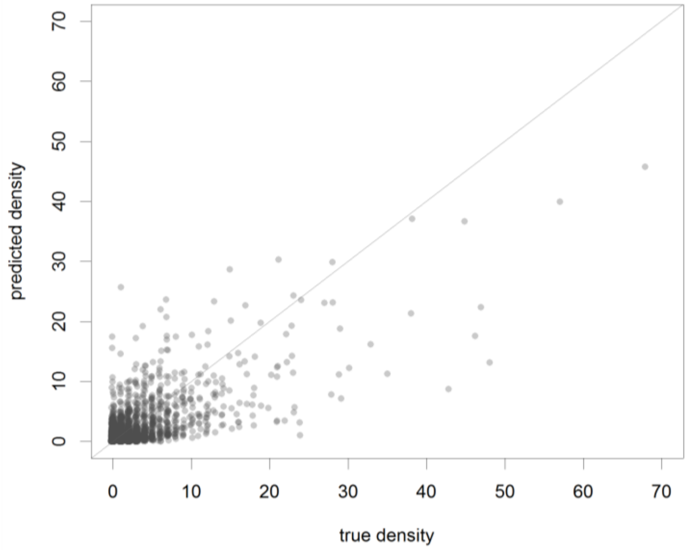


**Supplementary Figure 12. Predicted and true density of live-poultry markets for all pixels in China.** The diagonal line gives the theoretical perfect prediction. A small amount of noise was added to the true densities to aid visualisation where points overlap.

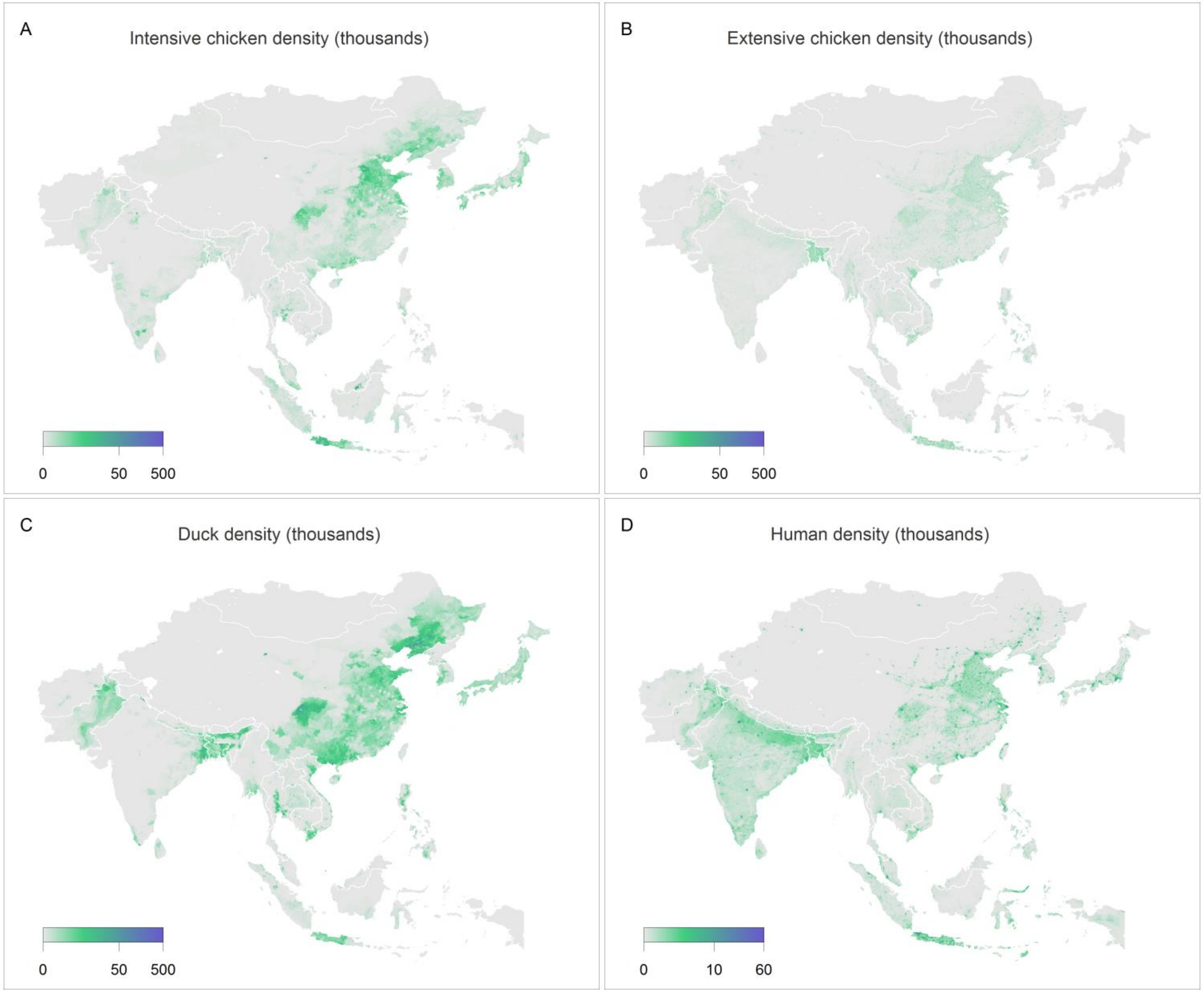


**Supplementary Figure 13 A-D. Distributions of environmental covariates used to predict risk of H7N9 infection across Asia.** Poultry and human densities are given as the number per square kilometre. To aid visualisation, all covariates are plotted on a $log_{10}(x + 1)$ scale.

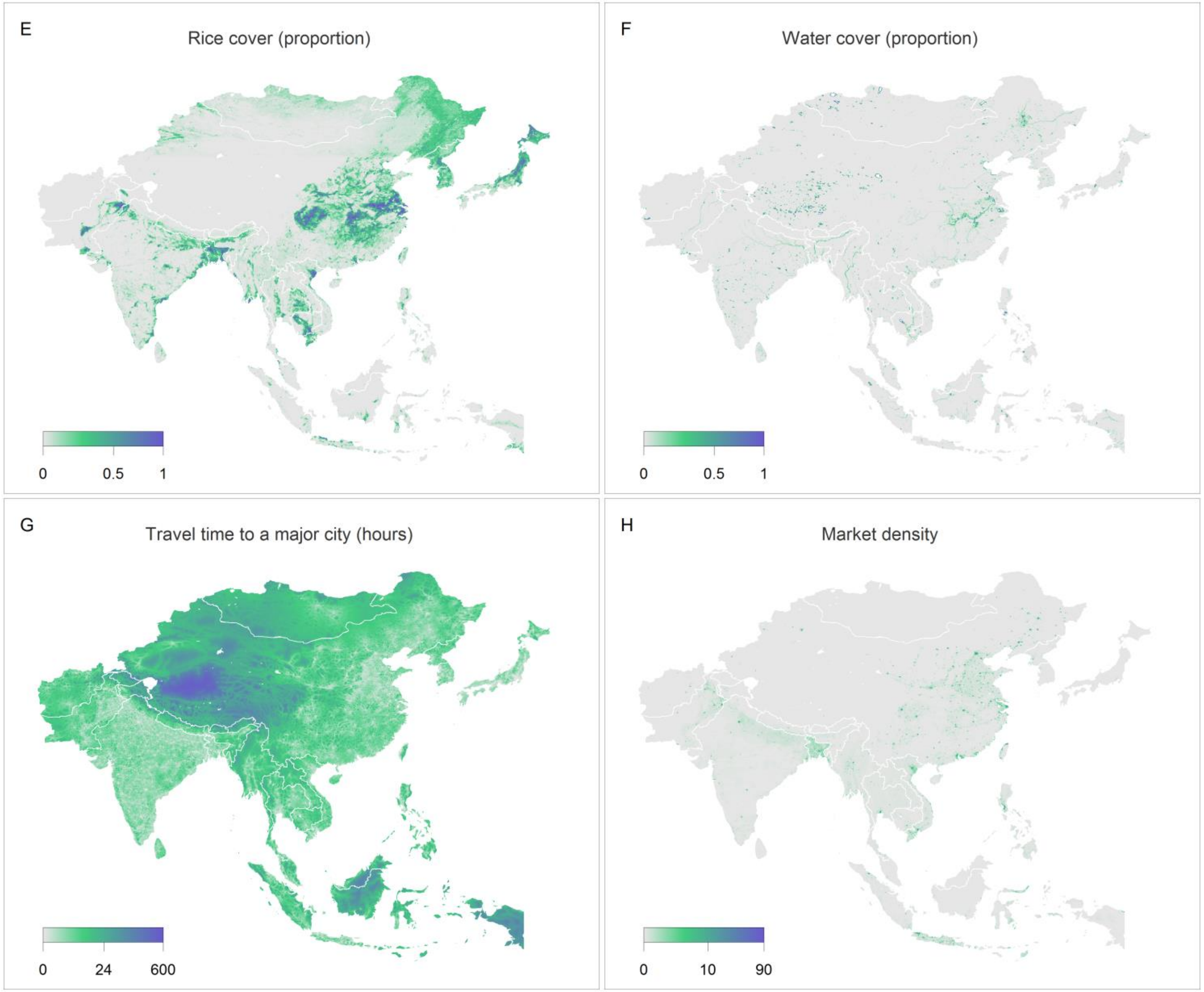


**Supplementary Figure 13 E-H. Distributions of environmental covariates used to predict risk of H7N9 infection across Asia.** Market density (H) is given as the number of markets per pixel. To aid visualisation, all covariates are plotted on a $log_{10}(x + 1)$ scale.

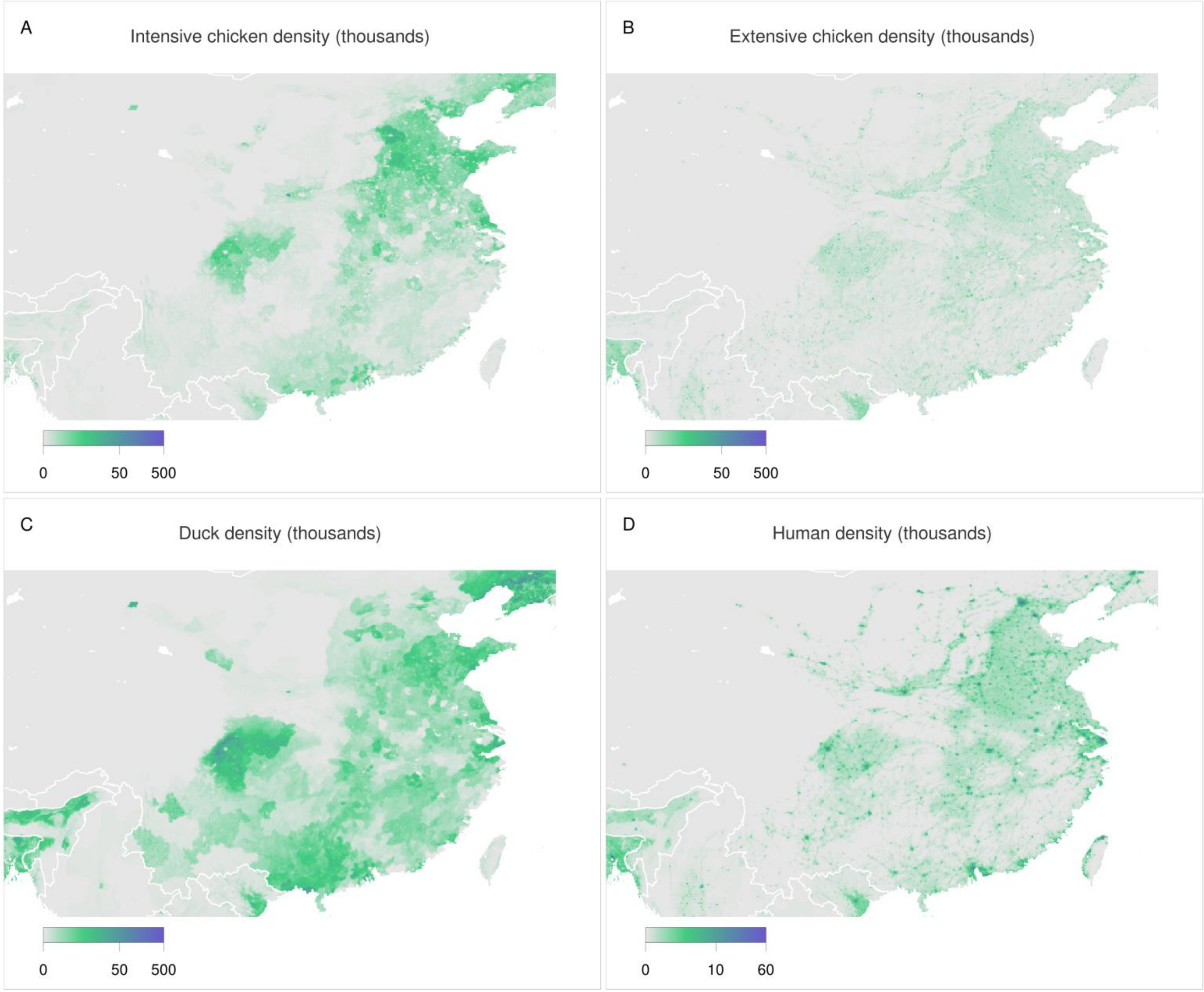


**Supplementary Figure 14 A-D. Distributions of environmental covariates used to predict risk of H7N9 infection in eastern China.** Poultry and human densities are given as the number per square kilometre. To aid visualisation, all covariates are plotted on a $log_{10}(x+1)$ scale.

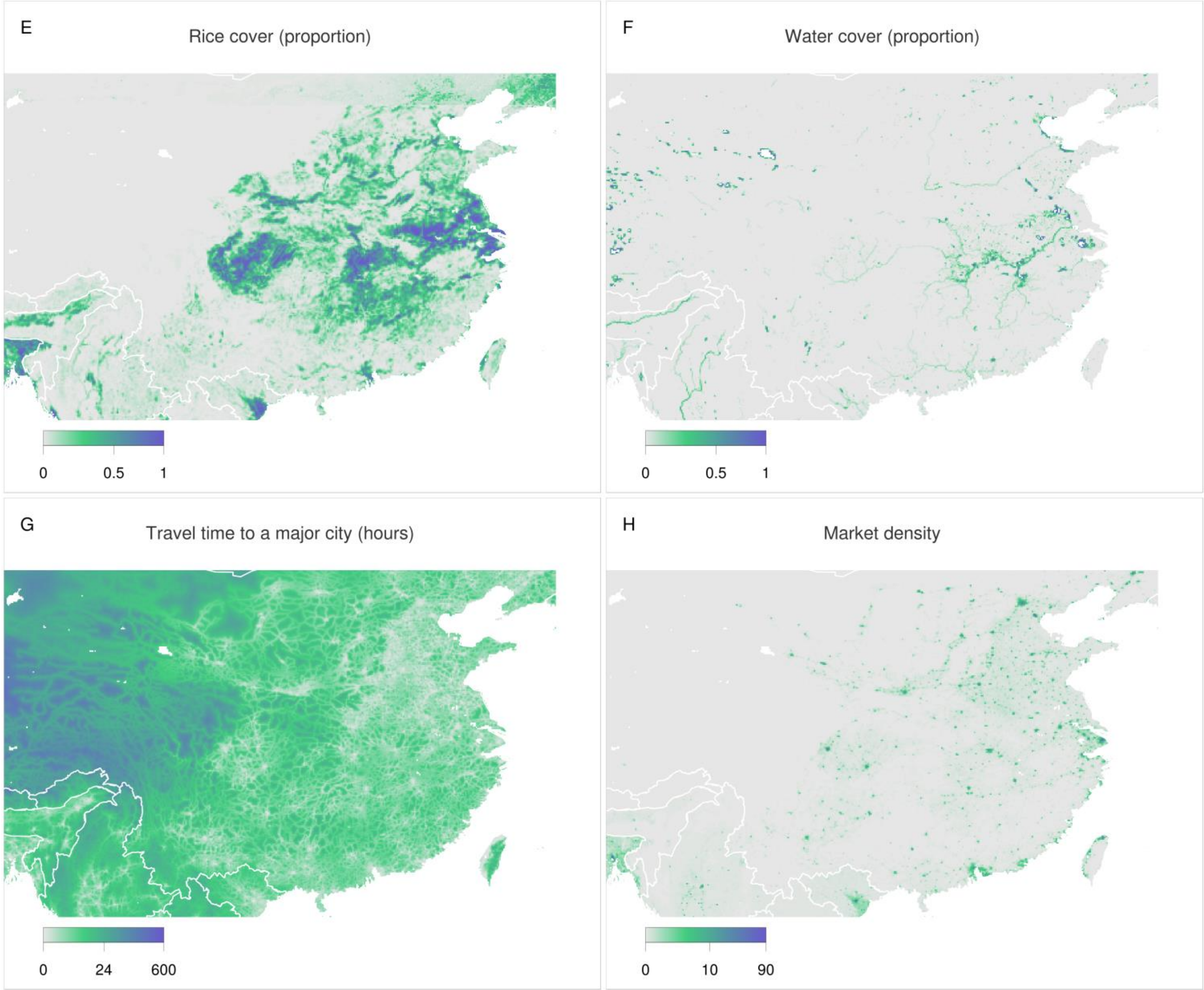


**Supplementary Figure 14 E-H. Distributions of environmental covariates used to predict risk of H7N9 infection in eastern China.** Market density (H) is given as the number of markets per pixel. To aid visualisation, all covariates are plotted on a $log_{10}(x+1)$ scale.

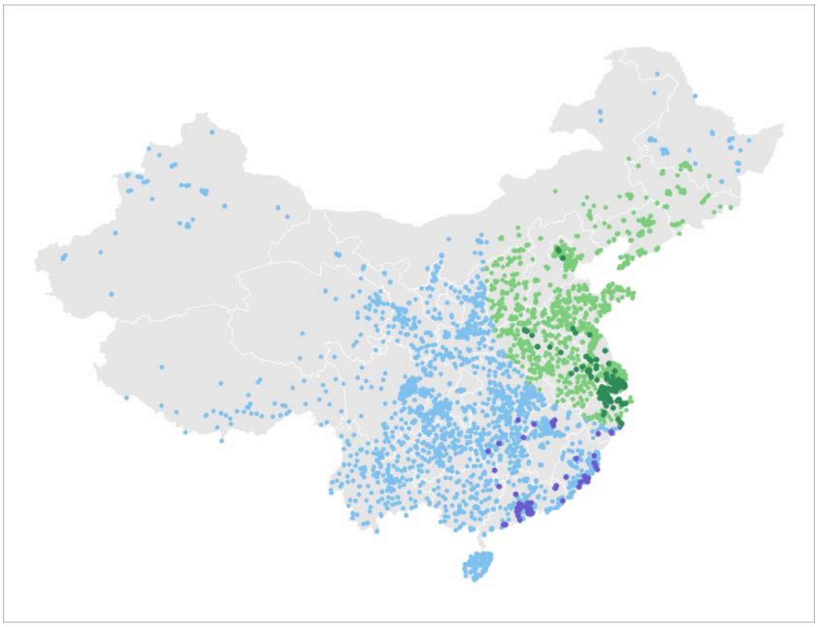

**Supplementary Figure 15. Illustration of the disc-based geographic stratification used to evaluate the extrapolation capacity of H7N9 market-level risk models.** The blue points give the locations of live-poultry markets used to train the model (dark blue points are H7N9 positive, light blue are negative) and green points give the locations of markets used to evaluate model fit (dark green points are H7N9 positive, light green are negative).

**Supplementary Table 1. Estimated number of extensively raised poultry and live-poultry markets in the countries of East, Southeast and South Asia.**

| Country | Chicken (millions) | Ducks (millions) | Poultry (millions) | Prop. Ext. | Ext. Poultry (millions) | N. markets |
|---|---|---|---|---|---|---|
| Afghanistan | 12.888 | 3.1412 | 16.0292 | 98.0% | 15.7 | 99.8 |
| Azerbaijan | 22.432 | 0.9188 | 23.3508 | 20.0% | 4.7 | 29.7 |
| Bangladesh | 228.035 | 42.677 | 270.712 | 81.0% | 219.3 | 1393.0 |
| Bhutan | 0.349 | 0.0726 | 0.4216 | 31.2% | 0.1 | 0.8 |
| Cambodia | 17.448 | 7 | 24.448 | 65.0% | 15.9 | 101.0 |
| China | 5200 | 789.569 | 5989.569 | 23.5% | 1408.7 | 8949.0 |
| Dem. People’s Rep of Korea | 16.569 | 5.936 | 22.505 | 58.6% | 13.2 | 83.8 |
| India | 841.865 | 26 | 867.865 | 43.0% | 373.2 | 2370.7 |
| Indonesia | 1349.626 | 45.292 | 1394.918 | 24.0% | 334.8 | 2126.8 |
| Japan | 286 | 12.6536 | 298.6536 | 1.4% | 4.1 | 26.3 |
| Lao People’s Democratic Republic | 25.105 | 3.2 | 28.305 | 84.0% | 23.8 | 151.0 |
| Malaysia | 225.79 | 48.2 | 273.99 | 1.0% | 2.7 | 17.4 |
| Mongolia | 0.426 | 0.2756 | 0.7016 | 37.4% | 0.3 | 1.7 |
| Myanmar | 156.407 | 12.6 | 169.007 | 84.0% | 142.0 | 901.9 |
| Nepal | 25.76 | 0.379 | 26.139 | 79.5% | 20.8 | 132.0 |
| Pakistan | 321 | 3.5 | 324.5 | 44.0% | 142.8 | 907.1 |
| Philippines | 158.984 | 10.268 | 169.252 | 75.0% | 126.9 | 806.4 |
| Republic of Korea | 149.2 | 14.397 | 163.597 | 1.0% | 1.6 | 10.4 |
| Sri Lanka | 14.018 | 0.013 | 14.031 | 15.0% | 2.1 | 13.4 |
| Thailand | 231.918 | 29.233 | 261.151 | 33.0% | 86.2 | 547.5 |
| Viet Nam | 218.201 | 68.633 | 286.834 | 70.0% | 200.8 | 1275.5 |

# SUPPLEMENTARY METHODS

## A) MARKET SURVEILLANCE AND LITERATURE SEARCH DATA

In addition to the human cases data collected by China CDC, a database of positive identification of influenza A (H7N9) in live-poultry markets was assembled, combining data from surveillance carried out by local CDC offices and the Ministry of Agriculture (MOA, http://www.moa.gov.cn/zwllm/yjgl/yqfb/), and with the records of influenza A (H7N9) published in scientific papers. Here, we summarize information obtained from MOA and the procedure used to extract data from scientific papers

*MOA surveillance*

Prior to the emergence of H7N9, the purpose of routine surveillance of poultry and poultry market in China was to detect highly pathogenic avian influenza. Routine surveillance consists in a combination of active surveillance and passive surveillance. Passive surveillance targets poultry and wild birds which are found dead of sickness or unknown apparent reasons, whereas active surveillance focuses on establishing fixed sentinels and randomly sampling the sentinels to monitor. The primary objectives of national surveillance are chicken, duck, goose and other poultry or wild birds. These are sampled from poultry farms, commercial poultry farms, backyard poultry raising households, poultry trading markets and slaughter houses as well as from main habitats of migratory birds for wild birds. Diagnosis methods include serological test by hemagglutination inhibition test (HI) (GB/T 18936-2003), as well as etiological test carried out through RT-PCR and fluorescent RT-PCR (GB/T 19438.2-2004). The active surveillance scheme is conducted twice a year and organized at the province level. The guidelines provided to all provinces for the serological surveillance are to organize surveillance through random sampling proportional to the number of birds in the farm. While for the etiological surveillance, the provinces are required to randomly select at least 120 epidemiological units (excluding villages) with at least 30 chicken or other poultry in each group. The results obtained at province level are hence submitted to the national level, and the national reference lab should report the test results every month. Since the emergence of H7N9 outbreak in China, the MOA issued an Emergency Surveillance Scheme on H7N9 on 7th August, 2013. The surveillance targets were specified chicken (especially layer, yellow feather broilers and other breeds which have long raising cycle), waterfowl (duck, goose), domestic pigeon and quail, wild birds and environment in high risk areas. The scope of surveillance was specified to be all poultry trading markets in China, stalls selling live poultry in farmers markets, poultry with certain size, backyard poultry raising farmers, poultry slaughter houses, and habitats of migratory birds. Since September 2013 onward (data used in this study only included epidemiological data collected until the $27^{th}$ Jan. 2014) , a centralized surveillance scheme was conducted. Every surveyed markets had to be sampled according to an assumed prevalence of 2%, by 150 throat and cloacal swabs and 150 serological samples. In addition, 30 environmental samples had to be tested. For the live poultry markets which tested H7N9 positive, 30 serological and etiological samples had to be prepared and positive results had to be reported to the national level as soon as possible.

*Literature search*

We used the key words of “H7N9” and “Poultry” or “live poultry market” or “live bird market” in PUBMED searches (http://www.ncbi.nlm.nih.gov/pubmed/ ) from March 31

onward, 2013 when Chinese government officially announced the confirmation of novel H7N9 virus to Feb 2, 2014. A total of 48 published papers were included at the first stage, and after reading, only 10 papers were included into our analysis because they include information positive tests for H7N9 by real time RT-PCR from samples either collected from poultry or live poultry markets [1–10].

## B) MAPPING HUMAN POPULATION DENSITY

Human population densities across the region were mapped primarily using datasets produced by the WorldPop project (www.worldpop.org.uk), and Gridded Population of the World (GPW)[11] for those countries where WorldPop data were unavailable. For the WorldPop datasets, census data at as high an administrative unit as available for the *circa* 2010 round of national censuses were assembled (see http://www.worldpop.org.uk/data/methods/ for full list of input census data). A novel semi-automated dasymetric modelling approach that incorporated the detailed census and ancillary data layers in a flexible random forest statistical model was then applied[12] to generate modelled gridded predictions of population density at approximately 100m spatial resolution. The use of the random forest technique in combination with covariate layers that include OpenStreetMap-derived infrastructure (http://www.openstreetmap.org/), Landsat-derived land cover data (http://www.mdafederal.com/geocover/geocoverlc/gclcoverview), satellite nightlights (http://ngdc.noaa.gov/eog/viirs/download_viirs_ntl.html), slope (http://hydrosheds.cr.usgs.gov/index.php), and a variety of settlement features[13], amongst others related to human population distributions, has been shown to produce substantial increases in population mapping accuracies over previous approaches examined[11,14]. This prediction layer was then used as the weighting surface to perform dasymetric redistribution of census counts at a country level to create a population count surface. Where census data were not available for 2010, UN derived urban and rural growth rates (http://esa.un.org/unup/) were applied to adjust counts. The final datasets were degraded to the spatial resolution of other layers used in the analyses. Where these WorldPop datasets were unavailable at the time of writing, GPW data adjusted to match 2010 UN population totals were incorporated.

## C) MAPPING CHICKEN AND DOMESTIC DUCK DENSITIES

Since the livestock densities of the Gridded Livestock of the World (GLW) were produced[15] significant improvements have been made in the sub-national statistics on reported poultry numbers; a new set of 1 km predictor variables, based on Fourier-processed MODIS imagery, has been compiled; and numerous modifications have been made to the modelling approach. Some of these improvements have already been applied to modelling poultry distributions in Asia[16,17] and new, global 1 km resolution datasets for all livestock species are currently being produced under a collaborative effort between UN-FAO, ILRI (under the CGIAR Research Programmes on the Humidtropics, CCAFS and A4NH), the Université Libre de Bruxelles, ERGO and others at the University of Oxford. These are freely available for download from the Livestock-Geo-Wiki (http://www.geo-wiki.org/branches/livestock/).

Supplementary Fig. 9 gives an overview of the livestock modelling process applied (which is described in more detail in Van Boeckel *et al.*[16]). The starting point is the collection of reported statistics on livestock numbers from many sources and for a range of years. The majority of the data originate from agricultural censuses or surveys carried out by

government departments but often the reported figures are estimated from those and compiled in statistical yearbooks. Data from the most recent years were compiled at the highest spatial resolution (smallest administrative areas) available. Metadata are available from the authors, and are provided with the GIS layers available through the Livestock-Geo-Wiki.

These reported statistics are then linked to a polygon file of administrative areas at the corresponding level. The resulting geographic database is then masked using a 1 km resolution raster grid to exclude areas not suitable for poultry production (e.g. dense urban areas, water bodies and protected areas). The effective density of livestock, accounting for the area suitable, is estimated for each polygon and assigned to each 1 km pixel, with unsuitable areas receiving a density of 0.

This pixel level dataset is used to train statistical models to predict livestock densities from a suite of environmental predictor variables. From this dataset, a number (typically 5) of subsampled datasets is generated by selecting pixels at random, subject to a set of rules to ensure that samples are representative of the dataset (maintaining an average number of sample points per 10,000 square km (typically 30) and ensuring that at least one point is assigned in each polygon). For each sample point in each subsample, data values are extracted for a) the density of the livestock type in question; b) the appropriate suitability masks; c) a number (typically 3 or 4) of stratification layers (described below); and d) the values of the predictor variables, all mapped at 1 km spatial resolution. Each subsample is then subjected to a five-fold cross-validation, with 75% of sample points used to train models and the remaining 25% used to test their goodness of fit. The five cross-validation folds for each of the five subsamples leads to a total of 25 bootstrap datasets for each livestock type.

Because the relationships between poultry densities and other spatial variables are likely to differ under varying conditions of agro-ecology, demographics and socio-economics, the analysis is spatially stratified. The stratification layers included are: a) an unsupervised clustering into 25 'eco-zones' based on the predictor variables[15]; b) the 11 classes defined by the Global Livestock Production Systems (GLPS) map version 5[18]; and c) a series of 13 biomes[19].

A comprehensive set of predictor variables allows the model to take advantage of relationships between livestock densities and climatic, environmental, demographic and topographic variables. The majority of these predictor variables are derived from temporal Fourier decomposition of MODIS satellite imagery products recorded at 8-day intervals between 2001 and 2008[20]. The resulting imagery captures seasonal characteristics of the environment (land surface temperatures and vegetation indices, for example). These predictors are augmented by gridded data on vegetation (length of growing period and timing of greening-up and senescence), human population density, infrastructure and topography.

Separate regression models for the log(x+1) transform of livestock densities are derived for each stratum of each stratification scheme employed. In situations where a stratum has insufficient sample points to create a robust model a general, un-stratified model is used for that tile. This creates, for each stratification scheme, a mosaic of predicted values from different regression models depending on the stratum. The quadratic form of each predictor variable is also provided to each model to enable the model to fit non-linear effects of covariates, giving a model of the form:

$$log(y+1) = a + b_1x_1 + c_1x_1^2 + b_2x_2 + c_2x_2^2 + \dots + b_nx_n + c_nx_n^2$$

The regression models for each bootstrap are applied to the predictor variables, using each stratification scheme.

For each bootstrap the regression models created under each stratification scheme are merged by selecting, pixel by pixel, the model from the stratification scheme that resulted in the lowest residual mean square error (RMSE). This creates a 'best RMSE composite' prediction for each bootstrap. The predicted log densities for the 25% of sample points reserved for testing the model fits are then compared for a) the un-stratified model; b) the best RMSE composite model; and c) the individual stratifications used. The best RMSE composite was used by default in most instances. However, if one of the stratification schemes is comparable to the composite from the best RMSE, the principles of parsimony are followed and the model is simplified by using the regression equations from that single stratification scheme.

Based on either an individual stratification or an aggregate prediction for each bootstrap the predicted log densities are then averaged across the 25 bootstraps to give the mean predicted log (x + 1) density of poultry in each pixel. In the post-processing stages these results are first de-transformed and then adjusted so that the totals for each administrative area match those of the reported statistics. For polygons for which no reported data were available the predicted densities are retained. The second post-processing stage is that of country correction, where the pixel values are further adjusted to match FAOSTAT national totals for a specified reference year (2006 in the previously available datasets, and 2010 in the revised datasets described in the present study).

For China, census data on chicken and duck numbers at the end of the calendar year 2010 and the numbers of individuals sold per year were obtained from three sources: a) published yearbooks, such as the China Animal Husbandry Yearbook, Statistic Yearbook of China or provincial yearbooks (e.g. http://data.stats.gov.cn/); b) the official website of the Ministry of Agriculture of China (http://english.agri.gov.cn/) and Agricultural Bureaus at province and prefecture level; c) contact with provincial Bureaus of Animal Husbandry, provincial Departments of Commerce, Statistics Bureaus and Chinese Agricultural Universities to obtain any data not available from sources a or b. These data were combined with the data compiled by FAO for the other countries in Asia, and the modelling procedure described above was carried out with this hybrid data set.

## D) EXTENSIVELY AND INTENSIVELY RAISED CHICKEN

The next step involves disaggregating chicken densities between extensive and intensive production systems. Previous models of intensive versus extensive livestock production[18] estimated the proportion of extensively raised poultry (PExt) as a function of the output/input (O/I) ratio, where output was the amount of meat (in this case) produced in a year and input was the standing stock during the same period. During exploratory investigations *per capita* gross domestic product (GDP) (in Purchasing Power Parity (PPP), reference year 2006) was found to correlate strongly with O/I ratios (r = 0.81). Furthermore, national estimates of *per capita* GDP are available for most years and countries from the World Bank, and sub-national estimates are available for a number of countries. For the present analysis, PExt was modelled for chickens using log *per capita* GDP. We checked that this choice would not reduce predictive power, compared to the O/I ratio previously used, by simultaneously testing *per capita* GDP and O/I ratio in a multiple regression model to predict chicken PExt. It was found that the increase in predictability due to the inclusion of O/I ratio was marginal. A logit

link function was used to model the proportion of extensively-raised stock so that the predicted proportions were bounded between 0 and 1. The model was weighted by the stock, so that countries with greater livestock populations would have a stronger influence on the model than those with fewer. The final model for chickens and the goodness of fit plot are presented in Figure D.1. The model formulation was as follows:

$$PExt \ = \ 1 - [expr * (GDPYLG - L) \ / \ (1 + expr * (GDPYLG - L))]$$

where PExt is the proportion of extensively-raised chicken in a country, GDPYLG is the log10 -transformed *per capita* GDP in the same year as the PExt estimate (PPT 2006), and r and L are model parameters. The best-fit parameters identified by the non-linear, weighted least-square regression were $r$ = 3.735 and $L$ = 3.348 (supplementary Fig. 10). The correlation coefficient between observed and predicted *PExt* values was 0.806. This model was applied to all countries in the study area, using FAOSTAT national totals to predict the total stock of extensively raised chicken and ducks.

The final step is to distribute spatially the numbers of extensively-raised chickens (based either on reported statistics or estimated from the LGP model). The general approach described in Gilbert et al.[21] and Robinson et al.[18] was used, whereby extensively-raised chickens were distributed equally among the rural population.

The spatial disaggregation of extensively-raised animals involves: a) estimating the rural population in each country; b) estimating the total number of chickens raised extensively in each country based on reported (when available) or modelled PExt, multiplied by the total chicken population (taken from FAOSTAT 2006); c) combining (a) and (b) to estimate the average number of extensively-raised chickens per rural person for each country; and d) applying this rate to the mapped rural population density to estimate the number of extensively-raised chickens per pixel. The distribution of intensively-raised chickens is then estimated by subtracting the raster distribution of extensively-raised chickens from the modelled raster distribution of the total number of chickens. In some locations, the number of extensively-raised chickens is higher than the predicted total numbers; typically in situations where either a very high rural population density results in large predicted numbers of extensively-raised chickens, or if the predicted total density is very low. In these cases, the numbers of extensively-raised livestock are set to zero, and the numbers of extensively raised livestock in other pixels are corrected, pro rata, such that the national totals of numbers of livestock raised extensively and intensively match those reported or predicted.

## E) MAPPING LIVE-POULTRY MARKETS IN ASIA

Since the dataset of live-poultry markets was available only for China, a statistical model was fitted to this dataset and used to predict the number of live-poultry markets per pixel for all of Asia. To account for social and geographic differences in the abundance of live-poultry markets these predictions were subsequently corrected at a national level so that the predicted ratio of poultry markets to humans for each country matched the ratio of chickens to humans from recent census data.

For each of the 138,602 pixels covering mainland China, the number of live-poultry markets from the dataset within that pixel was calculated. The number of live-poultry markets was then modelled in a statistical framework.

Generalized additive models (GAMs) were fitted using the bam function (an implementation of GAM tailored for large datasets) in the R package mgcv[22] with a Poisson likelihood and a log-link. This implementation calculates an optimal degree of smoothing for each model term as a part of the model fitting procedure. A GAM was used since it is able to capture non-linear effects of covariates (though not non-additive interactions) as with a boosted regression tree model, but with much less computational burden. Additionally, the effectiveness of boosted regression tree models for modelling count data has not been assessed.

Four covariates were assessed for their capacity to predict the number of live-poultry markets per pixel: accessibility, density of ducks, density of chickens and human population density. The human population density data layer was obtained from the AsiaPop[14] database in all countries where it was available to date, and from the Gridded Population of the World (GPW) database[23] elsewhere. The travel time to major cities was extracted from the Nelson accessibility maps[24] and the poultry covariates are described in detail in Supplementary information A. All covariates were transformed prior to model fitting and smooth terms modelled using cubic regression splines.

To determine the optimal set of covariates to use in the final market model, the full model (using all covariates) was compared with models excluding each of these variables in turn and with models using each variable alone. Goodness of fit was compared using the Bayesian information criterion (BIC), which trades off goodness of fit against the complexity in the model in order to prevent overfitting. The model containing all terms had the best fit, with a BIC 12 lower than the second best model (which had all covariates except the accessibility covariate).

The market dataset distinguished between wholesale and resale markets. In order to assess whether these markets differed in their distributions, an second model was produced by separately fitting GAMs for the wholesale and retail market datasets and summing the predicted number of markets. Despite this alternative model having twice as many parameters, the fitted likelihood was no better than the initial model. This indicates that the spatial distribution of these two types of markets is very similar and that they differ only in their total number. The initial model (using data on the number of either type of market) was therefore used in subsequent analyses.

The smooth components of the final model are shown in supplementary Fig. 11. The final model explained 69.1% of the deviance in dataset (compared with an intercept-only null model). A plot of the true and predicted market densities for the training dataset (supplementary Fig. 12) showed no evidence of zero-inflation and the Chi-squared estimator of overdispersion indicated that the model residuals were not overdispersed, supporting the use of the Poisson likelihood.

This model was then used to calculate the expected number of live-poultry markets per pixel for the rest of Asia.

Whilst the spatial distribution of markets is likely to follow a similar pattern across Asia as in China, the average density of markets is likely to vary between regions, with some countries having more live-poultry markets per capita than others. At the country level, this is influenced by a) the average consumption of poultry per-capita (e.g. China, which has 4.3 chickens per person is likely to have more live-poultry markets per person than India, which has only 0.68 chickens per person), and b) by the proportion of poultry that gets traded through live-poultry markets, which was assumed to be correlated to the proportion of extensively-raised poultry. To illustrate this; Thailand, South Korea and Myanmar have

similar numbers of poultry per person, but the share of these poultry being traded through live-poultry markets is likely to be much higher in Myanmar, where a very high proportion of poultry is still produced under extensive modes of production and trade. To account for these national differences, the number of live-poultry markets per country was assumed to be proportional to the amount of extensively raised poultry (estimated as described above).

The number of live-poultry markets per 1,000,000 extensively raised chickens and ducks observed in China was then applied to other countries to estimate the total number of live-poultry markets in each country. The results of this procedure are presented in supplementary Table 1, and these totals were used to apply post-hoc national corrections to the predictions of the live-poultry market model.

## F) ENVIRONMENTAL COVARIATES

In order to predict H7N9 infection risk across Asia, a range of contemporary gridded environmental covariate raster layers were produced. These layers were either produced by updating existing datasets with new information or were built from scratch. The distributions of the 8 covariates are shown across Asia in supplementary Fig. 13 and in the area of eastern China from cases have been reported in supplementary Fig. 14.

## G) MODELLING AND EVALUATION

The results of two types of BRT models are presented in the main text: a pixel-level presence-background model and market-level presence-absence models using the values of environmental covariates aggregated across the area surrounding each market. This section provides additional information about the procedures used to fit these models.

### Pixel-level presence-background BRT model

In order to facilitate comparison of our modelling approach with those previously used to map avian influenza, we fitted a model to a dataset of the presence or assumed absence of H7N9 at the pixel-level in China. In order to fit this model, the values of environmental covariates were extracted at pixels in which infected markets were present (presence pixels) and at 5000 pixels randomly selected from across China (background or pseudo-absence pixels). We followed the methodology of Martin *et al.*[25], selecting background pixels only from areas of China at least 0.0833 decimal degrees from any pixel containing an infected market, and where the human population density was at least one.

This approach is widely used in the ecological literature for mapping the distributions of species where only sites of occurrence of the species are known[26]. There are several limitations of this approach. These include the ‘contamination’ of the pseudo-absence records (which are assumed by the statistical model to be sites of true absence) with sites in which the species (or in our case pathogen) is in fact present[27] and bias of the resulting predictions due to conflation of the true distribution of the species with the distribution of reporting probability[28]. In the case of H7N9 infection, which is closely tied to live-poultry markets and humans, this approach leads to a model which predicts the combined distribution of the

pathogen and the distributions of these reporting units. Since the distributions of markets and humans are easily predicted using environmental covariates, such models unsurprisingly lead to very high validation statistics[29]. Whilst models fitted in this way are useful for mapping risk at these spatial units, they lack a transparent biological interpretation regarding the ecology of the pathogen.

**Market-level presence-absence BRT models**

For the reasons stated above, we focused our analysis on live-poultry markets as the units of reporting. Instead of fitting BRT models using the values of environmental covariates at pixels in which infections had or had not been reported, we extracted the values of covariates corresponding to the location of each market, and trained the model to predict the probability that the market itself was infected. In order to account for the aggregating effect of markets, which import poultry from an area surrounding the market, we calculated the value of covariates at each market as a weighted mean of the values of covariates at pixels in an area surrounding the market. This weighting was calculated using an isotropic two-dimensional Gaussian smoothing kernel in geographic space, of the form:

$$e^{-\frac{(\boldsymbol{\mu}-\boldsymbol{x})^2}{2\sigma^2}}$$

where $\boldsymbol{x}$ are the coordinates of the pixel where the weighting is to be calculated, $\boldsymbol{\mu}$ are the coordinates of the market and $\sigma$ is a distance specified to represent the size of the market catchment area. All pixels within $5\sigma$ of the market in question were considered in the weighting. As well as training models using market-level covariate values aggregated in this way, continuous prediction maps of market-level risk were produced by making predictions to gridded surfaces with this weighted averaging procedure applied to all pixels.

**Model validation procedures**

Three types of model validation statistics are presented in the main text: training-set validation statistics, standard cross-validation statistics and spatially-stratified cross-validation statistics. Whilst a range of different validation statistics can be used for binary data such as the presence or absence of a pathogen, most of these are subject to an assumption that the *absolute* probability of presence can be accurately predicted[30]. This assumption is violated in the case of an emerging disease since the disease has yet to become established in all areas which are suitable for it. A commonly used metric in the species distribution modelling literature – the area under the receiver operating curve (AUC), instead evaluates the models ability to rank sites by probability of presence and is therefore the most suitable for this application.

Training-set and standard cross-validation AUCs were calculated as the mean of the scores of each of the 120 BRT submodels included in the ensemble. For each of these submodels the training-set AUC was calculated from the predictions to the full dataset used to train the model. Standard cross-validation AUCs were calculated using the four randomly-selected folds of the training datasets used to perform the final step of the cross-validation procedure of Elith et al.[31] which is used to fit the BRT submodel, whilst determining the optimal number of regression trees to include in the submodel.

Training-set validation statistics are the least reliable estimates of predictive capacity to a new dataset, since it is most prone to statistical overfitting. It is included here only to enable comparison with previous studies which have used this metric. Standard cross-validation is widely used to overcome this issue[32]. However, because training and evaluation records are

selected at random from the dataset, and occurrence records of a species or pathogen are spatially clustered, even a model with poor predictive ability may appear to predict well when measured in this way. Instead, the ability of a model to make accurate predictions in new locations is better measured by performing a spatially-stratified cross-validation where training and test sets are sampled from geographically distinct regions[33].

We carried out spatially-stratified cross-validation by assigning markets to either the training or evaluation datasets according to whether they fell outside (training) or inside (evaluation) a disc of radius 1000 km (supplementary Fig. 15). Discs were placed at random, centered on the location of a market, subject to the constraint that at least 45 infected markets (around 28% of the total number) were present in both the training and evaluation sets. This constraint ensured that sufficient data were available to adequately train the model and to evaluate its predictive capacity. The disc-fold validation procedure was implemented in R[34] using code adapted from the sperrorest package[35]. This disc-fold validation procedure was repeated 5 times for each model run (example in supplementary Fig. 15), with a full BRT ensemble fitted for each fold, and the mean and standard error of the AUCs calculated.

**Collinearity of environmental covariates**

Many of the covariate layers used in this analysis exhibit similar spatial distributions across China and the rest of Asia. In addition, a number of these have been constructed by combining other environmental layers (such as human population density) with additional datasets. Whilst all of these environmental layers are considered to be risk factors for avian influenza infection, they exhibit high collinearity. Collinearity can lead to problems with fitting many commonly used statistical models (such as generalized linear and additive models), leading to poor predictions[36]. Because the boosted regression tree (BRT) approach we apply is able to fit complex (i.e. non-additive) functions in the environmental space, the accuracy of its predictions are unaffected by collinearity in environmental covariates[31]. This is one of the reasons that this approach has been so successful at predictive modelling of the distributions of species and diseases[37,38]. However, whilst predictions from BRT models are unaffected, such collinearity does limit our ability to test eco-epidemiological hypotheses about the drivers of H7N9 distribution. As such, the ordering of the relative contributions of environmental covariates (as displayed in Figure 2 in the main text) should be treated only as an indication of likely importance of groups of covariates, rather than a formal comparison.

## SUPPLEMENTARY REFERENCES


1. Yang, P. *et al.* A case of avian influenza A (H7N9) virus occurring in the summer season, China. *J. Infect.* **67,** 624–625 (2013).

2. Wen, Y.-M. & Klenk, H.-D. H7N9 avian influenza virus - search and re-search. *Emerg. Microbes Infect.* **2,** e18 (2013).

3. Shi, J. *et al.* A detailed epidemiological and clinical description of 6 human cases of avian-origin influenza A (H7N9) virus infection in Shanghai. *PloS One* **8,** e77651 (2013).

4. Yang, P. *et al.* Surveillance for avian influenza A(H7N9), Beijing, China, 2013. *Emerg. Infect. Dis.* **19,** 2041–2043 (2013).

5. Yang, F. *et al.* A fatal case caused by novel H7N9 avian influenza A virus in China. *Emerg. Microbes Infect.* **2,** e19 (2013).

6. Lee, S. S., Wong, N. S. & Leung, C. C. Exposure to avian influenza H7N9 in farms and wet markets. *Lancet* **381,** 1815 (2013).

7. Ip, D. K. M. *et al.* Detection of mild to moderate influenza A/H7N9 infection by China's national sentinel surveillance system for influenza-like illness: case series. *BMJ* **346,** f3693 (2013).

8. Han, J. *et al.* Clinical presentation and sequence analyses of HA and NA antigens of the novel H7N9 viruses. *Emerg. Microbes Infect.* **2,** e23 (2013).

9. Feng, Y. *et al.* Origin and characteristics of internal genes affect infectivity of the novel avian-origin influenza A (H7N9) virus. *PloS One* **8,** e81136 (2013).

10. Chen, Y. *et al.* Human infections with the emerging avian influenza A H7N9 virus from wet market poultry: clinical analysis and characterisation of viral genome. *Lancet* **381,** 1916–1925 (2013).

11. Balk, D. L. *et al.* Determining global population distribution: methods, applications and data. *Adv Parasitol* **62,** 119–156 (2006).

12. Stevens, F. R., Gaughan, A. E., Linard, C. & Tatem, A. J. Disaggregating census data for population mapping using Random Forests with remotely-sensed and other ancillary data. *PLos ONE* (In Press).

13. Schneider, A., Friedl, M. A. & Potere, D. Mapping global urban areas using MODIS 500-m data: New methods and datasets based on 'urban ecoregions'. *Remote Sens. Environ.* **114,** 1733–1746 (2010).

14. Gaughan, A. E., Stevens, F. R., Linard, C., Jia, P. & Tatem, A. J. High resolution population distribution maps for Southeast Asia in 2010 and 2015. *PloS One* **8,** e55882 (2013).

15. Wint, W., Robinson, T. P., Food & Nations, A. O. of the U. *Gridded Livestock of the World*. (Food and Agriculture Organization of the United Nations, 2007).

16. Van Boeckel, T. P. *et al.* Modelling the distribution of domestic ducks in Monsoon Asia. *Agric. Ecosyst. Environ.* **141,** 373–380 (2011).

17. Prosser, D. J. *et al.* Modelling the distribution of chickens, ducks, and geese in China. *Agric. Ecosyst. Environ.* **141,** 381–389 (2011).

18. Robinson, T. *et al.* Global livestock production systems. 152 pp. (2011).

19. Olson, D. M. *et al.* Terrestrial Ecoregions of the World: A New Map of Life on Earth: A new global map of terrestrial ecoregions provides an innovative tool for conserving biodiversity. *BioScience* **51,** 933–938 (2001).

20. Scharlemann, J. P. *et al.* Global data for ecology and epidemiology: a novel algorithm for temporal Fourier processing MODIS data. *PLoS One* **3,** e1408 (2008).

21. Gilbert, M., Wint, W. & Slingenbergh, J. *Ecological factors in disease emergence from animal reservoir*. 42 (Food and Agriculture Organizattion, 2004).

22. Wood, S. N. Fast stable restricted maximum likelihood and marginal likelihood estimation of semiparametric generalized linear models. *J. R. Stat. Soc. Ser. B Stat. Methodol.* **73,** 3–36 (2011).

23. Balk, D. L. *et al.* in *Adv. Parasitol.* **62,** 119–156 (Elsevier, 2006).

24. Nelson, A. *Travel time to major cities: A global map of Accessibility. Global Environment Monitoring Unit—Joint Research Centre of the European Commission, Ispra, Italy*. (2008).

25. Martin, V. *et al.* Spatial distribution and risk factors of highly pathogenic avian influenza (HPAI) H5N1 in China. *PLoS Pathog.* **7,** e1001308 (2011).

26. Elith, J. & Leathwick, J. R. Species Distribution Models: Ecological Explanation and Prediction Across Space and Time. *Annu. Rev. Ecol. Evol. Syst.* **40,** 677–697 (2009).

27. Ward, G. Statistics in ecological modeling; presence-only data and boosted mars. (2007). at <http://citeseerx.ist.psu.edu/viewdoc/download?doi=10.1.1.126.5750&rep=rep1&type=pdf>

28. Phillips, S. J. *et al.* Sample selection bias and presence-only distribution models: implications for background and pseudo-absence data. *Ecol. Appl.* **19,** 181–197 (2009).

29. Fang, L.-Q. *et al.* Mapping Spread and Risk of Avian Influenza A (H7N9) in China. *Sci. Rep.* **3,** (2013).

30. Lawson, C. R., Hodgson, J. A., Wilson, R. J. & Richards, S. A. Prevalence, thresholds and the performance of presence–absence models. *Methods Ecol. Evol.* **5,** 54–64 (2014).

31. Elith, J., Leathwick, J. R. & Hastie, T. A working guide to boosted regression trees. *J. Anim. Ecol.* **77,** 802–813 (2008).

32. Hastie, T. *et al. The elements of statistical learning*. **2,** (Springer, 2009).

33. Bahn, V. & McGill, B. J. Testing the predictive performance of distribution models. *Oikos* **122,** 321–331 (2013).

34. R. Core Team. *R: A Language and Environment for Statistical Computing. R Foundation for Statistical Computing, Vienna, Austria, 2012*. (ISBN 3-900051-07-0, 2012).

35. Brenning, A. Spatial cross-validation and bootstrap for the assessment of prediction rules in remote sensing: The R package sperrorest. in *Geosci. Remote Sens. Symp. IGARSS 2012 IEEE Int.* 5372–5375 (2012). doi:10.1109/IGARSS.2012.6352393

36. Dormann, C. F., Purschke, O., García Márquez, J. R., Lautenbach, S. & Schröder, B. Components of uncertainty in species distribution analysis: a case study of the Great Grey Shrike. *Ecology* **89,** 3371–3386 (2008).

37. Elith, J. *et al.* Novel methods improve prediction of species' distributions from occurrence data. *Ecography* **29,** 129–151 (2006).

38. Bhatt, S. *et al.* The global distribution and burden of dengue. *Nature* **496,** 504–507 (2013).
